# White matter microstructure and hyperintensity burden in isolated REM sleep behaviour disorder

**DOI:** 10.1093/braincomms/fcag414

**Published:** 2026-11-02

**Authors:** Elijah Mak, Scott A Przybelski, Robert I Reid, Angela J Fought, Sheelakumari Raghavan, Christopher G Schwarz, Prashanthi Vemuri, Clifford R Jack, Yuzheng Nie, Hernis De La Cruz, Alon Y Avidan, Donald L Bliwise, Meghan C Campbell, Susan Criswell, Albert A Davis, Kevin Duff, Kaylena A Ehgoetz Martens, Jonathan E Elliott, Tanis J Ferman, Julie A Fields, Leah K Forsberg, Jean-François Gagnon, Ziv Gan-Or, Michael J Howell, Michele T M Hu, Xiaoping Hu, Daniel E Huddleston, Paul T Kotzbauer, Jason Langley, Miranda M Lim, Jessica Locke, Val J Lowe, Stuart J McCarter, Jennifer S McLeland, Mitchell Miglis, Emmanuel Mignot, Toji Miyagawa, Lee E Neilson, Kendall James Nichols, Amélie Pelletier, Owen A Ross, Carlos H Schenck, Wolfgang Singer, Erik K St. Louis, Lynn Marie Trotti, Aleksandar Videnovic, Chengjie Xiong, Ronald B Postuma, Yo-El Ju, Bradley F Boeve, Kejal Kantarci

**Affiliations:** Department of Radiology, Mayo Clinic, Rochester, MN 55905, USA; Department of Quantitative Health Sciences, Mayo Clinic, Rochester, MN 55905, USA; Department of Information Technology, Mayo Clinic, Rochester, MN 55905, USA; Department of Quantitative Health Sciences, Mayo Clinic, Rochester, MN 55905, USA; Department of Radiology, Mayo Clinic, Rochester, MN 55905, USA; Department of Radiology, Mayo Clinic, Rochester, MN 55905, USA; Department of Radiology, Mayo Clinic, Rochester, MN 55905, USA; Department of Radiology, Mayo Clinic, Rochester, MN 55905, USA; Center for Biostatistics and Data Science, Washington University School of Medicine, St. Louis, MO 63110, USA; Center for Biostatistics and Data Science, Washington University School of Medicine, St. Louis, MO 63110, USA; Department of Neurology, David Geffen School of Medicine, University of California at Los Angeles, Los Angeles, CA 90095, USA; Department of Neurology, Emory University School of Medicine, Atlanta, GA 30322, USA; Department of Neurology, Washington University School of Medicine, St. Louis, MO 63110, USA; Department of Neurology, Barrow Neurological Institute, Phoenix, AZ 85013, USA; Department of Neurology, Washington University School of Medicine, St. Louis, MO 63110, USA; Department of Neurology, Oregon Health & Science University, Portland, OR 97239, USA; Department of Kinesiology and Health Sciences, University of Waterloo, Waterloo, ON N2L 3G1, Canada; Department of Neurology, Oregon Health & Science University, Portland, OR 97239, USA; Research Service, VA Portland Health Care System, Portland, OR 97239, USA; Department of Psychiatry and Psychology, Mayo Clinic, Jacksonville, FL 32224, USA; Department of Psychiatry and Psychology, Mayo Clinic, Rochester, MN 55905, USA; Department of Neurology, Mayo Clinic, Rochester, MN 55905, USA; Department of Psychology, Université du Québec à Montréal, Montreal, QC H3C 3P8, Canada; Department of Neurology and Neurosurgery, The Neuro (Montreal Neurological Institute-Hospital), McGill University, Montreal, QC H3A 2B4, Canada; Department of Neurology, University of Minnesota, Minneapolis, MN 55455, USA; Nuffield Department of Clinical Neurosciences, University of Oxford, Oxford OX3 9DU, UK; Center for Advanced Neuroimaging, University of California, Riverside, Riverside, CA 92521, USA; Department of Neurology, Emory University School of Medicine, Atlanta, GA 30322, USA; Department of Neurology, Washington University School of Medicine, St. Louis, MO 63110, USA; Center for Advanced Neuroimaging, University of California, Riverside, Riverside, CA 92521, USA; Department of Neurology, Oregon Health & Science University, Portland, OR 97239, USA; Research Service, VA Portland Health Care System, Portland, OR 97239, USA; Department of Neurology, Oregon Health & Science University, Portland, OR 97239, USA; Department of Radiology, Mayo Clinic, Rochester, MN 55905, USA; Department of Neurology, Mayo Clinic, Rochester, MN 55905, USA; Department of Neurology, Washington University School of Medicine, St. Louis, MO 63110, USA; Department of Neurology and Neurological Sciences, Stanford University, Palo Alto, CA 94304, USA; Department of Psychiatry and Behavioral Sciences, Stanford University, Palo Alto, CA 94304, USA; Department of Neurology, Mayo Clinic, Rochester, MN 55905, USA; Department of Neurology, Oregon Health & Science University, Portland, OR 97239, USA; Research Service, VA Portland Health Care System, Portland, OR 97239, USA; Department of Neurology, Emory University School of Medicine, Atlanta, GA 30322, USA; Centre for Advanced Research in Sleep Medicine, Hôpital du Sacré-Cœur de Montréal, Montreal, QC H4J 1C5, Canada; Department of Neuroscience, Mayo Clinic, Jacksonville, FL 32224, USA; Department of Psychiatry, University of Minnesota, Minneapolis, MN 55455, USA; Department of Psychiatry, Minnesota Regional Sleep Disorders Center, Hennepin County Medical Center, Minneapolis, MN 55415, USA; Department of Neurology, Mayo Clinic, Rochester, MN 55905, USA; Department of Neurology, Mayo Clinic, Rochester, MN 55905, USA; Department of Neurology, Emory University School of Medicine, Atlanta, GA 30322, USA; Department of Neurology, Massachusetts General Hospital/Harvard Medical School, Boston, MA 02114, USA; Center for Biostatistics and Data Science, Washington University School of Medicine, St. Louis, MO 63110, USA; Department of Neurology and Neurosurgery, The Neuro (Montreal Neurological Institute-Hospital), McGill University, Montreal, QC H3A 2B4, Canada; Department of Neurology, Washington University School of Medicine, St. Louis, MO 63110, USA; Department of Neurology, Mayo Clinic, Rochester, MN 55905, USA; Department of Radiology, Mayo Clinic, Rochester, MN 55905, USA

**Keywords:** isolated REM sleep behaviour disorder, diffusion MRI, white matter hyperintensities, prodromal synucleinopathy, NODDI

## Abstract

Isolated rapid eye movement sleep behaviour disorder represents a prodromal phase of α-synucleinopathy. Diffusion MRI provides *in vivo* markers of white matter microstructural properties, while white matter hyperintensities reflect cerebrovascular injury. However, their relative contributions to cognitive and clinical dysfunctions in patients with isolated rapid eye movement sleep behaviour disorder remain unclear. We analysed 168 polysomnography-confirmed isolated rapid eye movement sleep behaviour disorder participants from the North American Prodromal Synucleinopathy Consortium. Multi-shell diffusion MRI was used to derive metrics from diffusion tensor imaging (fractional anisotropy and mean diffusivity) and Neurite Orientation Dispersion and Density Imaging (tissue-weighted neurite density index and orientation dispersion index) across 39 white matter regions using a modified Johns Hopkins University white matter atlas. White matter hyperintensity burden was quantified from fluid-attenuated inversion recovery MRI. Using linear regression adjusted for age and sex, we compared white matter microstructure and hyperintensity burden between clinically impaired and unimpaired participants and examined associations with multiple cognitive and clinical domains, including global cognition, working memory, motor severity, manual dexterity, autonomic dysfunction, olfactory function, prodromal disease severity and daytime sleepiness. False discovery rate (FDR) correction was applied to account for multiple comparisons. In our sample (*n* = 168), participants with clinical impairment (*n* = 57) demonstrated higher white matter hyperintensity burden than unimpaired participants (*n* = 111) and showed limited microstructural differences, including lower fractional anisotropy in the superior fronto-occipital fasciculus and inferior occipital white matter and higher orientation dispersion index in inferior occipital white matter. After adjusting for white matter hyperintensity burden, only increased occipital orientation dispersion index remained statistically significant. Diffusion metrics showed widespread associations with digit span forward performance and autonomic dysfunction. In contrast, increased white matter hyperintensity burden demonstrated broader associations with poorer Montreal Cognitive Assessment scores, poorer digit span forward performance, lower Purdue Pegboard performance and higher Prodromal Synucleinopathy Rating Scale scores, indicating greater prodromal disease severity. Our findings highlight cerebrovascular white matter burden as an important correlate of clinical dysfunction in isolated rapid eye movement sleep behaviour disorder and underscore the need to consider cerebrovascular lesions when interpreting diffusion imaging markers in prodromal synucleinopathy.

## Teaser

Mak *et al*. analysed diffusion and fluid-attenuated inversion recovery magnetic resonance imaging from 168 participants enrolled with polysomnography-confirmed isolated rapid eye movement sleep behaviour disorder. Age- and sex-adjusted regressions showed limited regional diffusion differences but greater white matter hyperintensity burden in participants with mild cognitive impairment or phenoconversion.

## Graphical Abstract

**Graphical Abstract:**
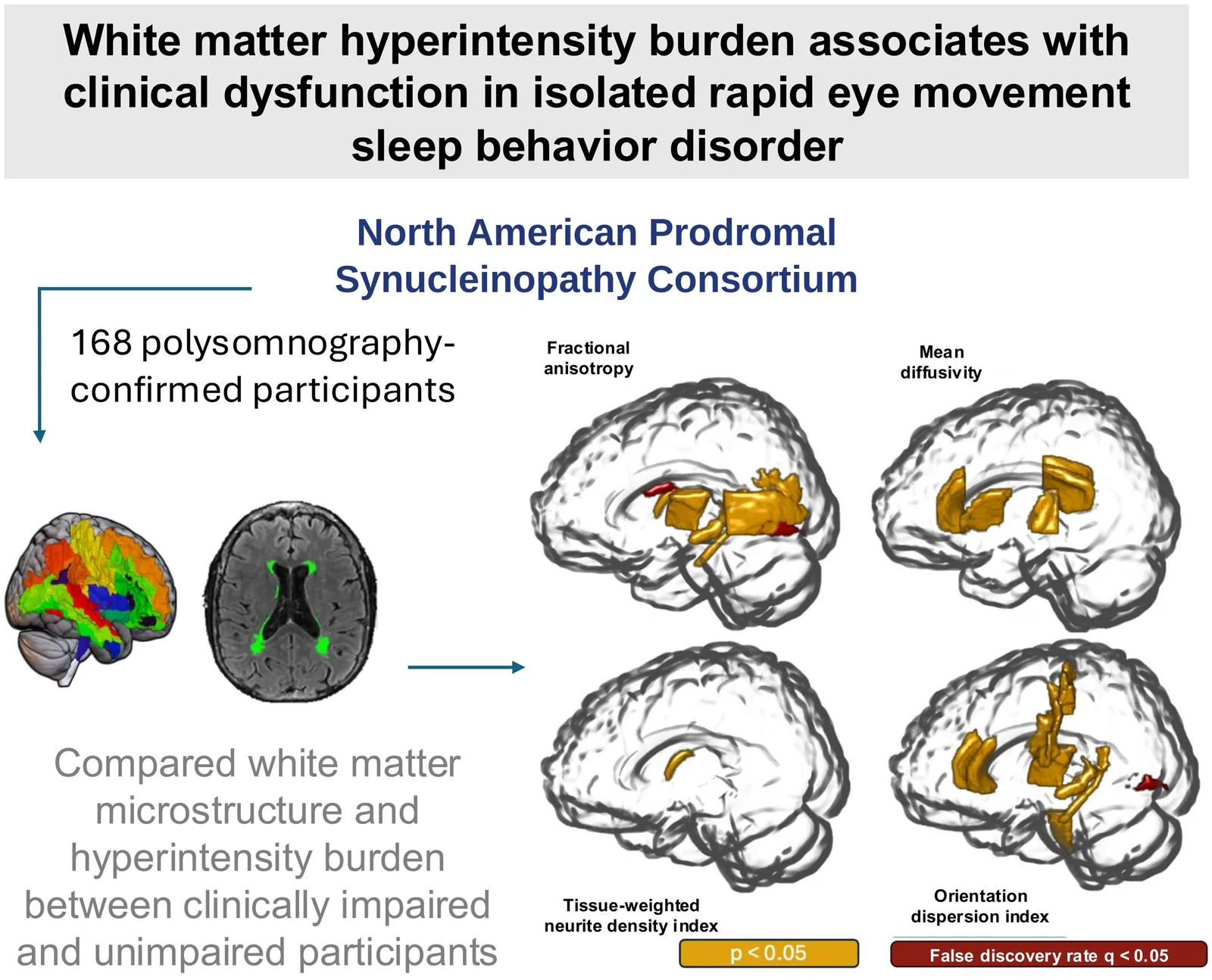

## Introduction

Isolated REM sleep behaviour disorder (iRBD) is a parasomnia marked by dream-enactment behaviour arising from loss of the muscle atonia that is normally present during REM sleep.^1,2^ More than 80% of older adults who are clinically diagnosed as iRBD patients will ultimately develop an overt neurodegenerative disease within 10 years of diagnosis, with annual phenoconversion rates of 6–8% and cumulative risk estimates of 40–65% after 10 years.^3-6^ This highlights the need for sensitive neuroimaging biomarkers that could capture early brain changes associated with underlying pathology and subsequent risks of clinical progression.^7^

To that end, growing evidence indicates that individuals with iRBD already exhibit widespread neurobiological alterations consistent with early Lewy body disease. Structural MRI studies have revealed grey matter atrophy^8,9^ while heterogeneous patterns of both hyper- and hypometabolism have been detected on FDG-PET imaging.^10^ Delineating the neural substrates underlying cognitive dysfunction in iRBD is therefore critical for stratifying conversion risk and identifying patients who may stand to benefit more from neuroprotective treatments before overt neurodegeneration.

In addition to structural MRI and FDG-PET investigations, diffusion MRI enables assessment of white matter integrity through metrics such as fractional anisotropy (FA) and mean diffusivity (MD). However, conventional diffusion tensor imaging (DTI) metrics reflect composite tissue properties and cannot disentangle neurite density from fibre architecture, particularly in regions with crossing fibres.^11^ Moreover, inconsistencies across prior iRBD studies may partly reflect the limited biological specificity of DTI metrics,^12,13^ which are influenced by multiple factors including axonal density, myelination, fibre orientation and extracellular water content. Neurite orientation dispersion and density imaging (NODDI) addresses this limitation through a biophysical multi-compartment model that separates diffusion signals into biologically meaningful compartments.^14^ The neurite density index (NDI) reflects intracellular volume fraction of axons and dendrites, the orientation dispersion index (ODI) quantifies fibre dispersion and architectural coherence and the isotropic volume fraction (ISOVF) captures free-water content potentially reflecting extracellular fluid or neuroinflammation. NODDI has been validated against histological markers in post-mortem human tissue^15^ and synaptic density measured from [^11^C]-UCB-J PET^16^ and has demonstrated sensitivity to microstructural alterations across several neurodegenerative conditions, including Alzheimer’s disease, Parkinson’s disease and DLB.^12,17-20^ Despite these advances, NODDI has not been systematically applied to examine white matter microstructure–clinical associations across multiple cognitive and motor domains in large iRBD cohorts.

Cerebrovascular pathology may represent an additional contributor to white matter injury that may act in concert with synucleinopathy-related processes and contribute to clinical heterogeneity in prodromal synucleinopathy.^21^ White matter hyperintensities (WMHs), a radiological marker of cerebral small vessel disease reflecting chronic ischaemia and demyelination, are frequently observed in older adults and are correlated with cognitive decline and motor deficits across several neurodegenerative disorders.^22-24^ Growing evidence suggests that vascular pathology may be present in iRBD, as diffusion-derived metrics of small vessel disease, including peak width of skeletonized mean diffusivity (PSMD), are increased compared to healthy controls, indicating coexisting cerebrovascular injury.^25^ However, the extent to which WMH burden relates to clinical manifestations in iRBD remains poorly understood.

Here, we combined DTI and NODDI metrics with quantitative WMH burden to examine white matter alterations in polysomnography-confirmed iRBD participants enrolled in the North American Prodromal Synucleinopathy (NAPS) Consortium (https://www.naps-rbd.org), a multi-centre initiative across North America designed to deeply phenotype a large cohort of iRBD patients through standardized clinical and biomarker assessments with ongoing annual longitudinal follow-up (Fig. 1).^26^ First, we compared white matter microstructure [FA, MD, tissue-weighted NDI (tNDI) and ODI] and WMH burden between iRBD participants with clinical impairment or phenoconversion to parkinsonism versus those who are unimpaired and conducted sensitivity analyses with additional adjustment for WMH burden to assess whether group differences in microstructure were attenuated after adjustment for WMH burden. Second, we examined associations between white matter metrics and WMH with a composite measure of prodromal disease severity (Prodromal Synucleinopathy Rating Scale, PSRS), alongside domain-specific measures of global cognition, working memory, motor severity, manual dexterity, autonomic dysfunction, olfactory function and daytime sleepiness.

**Figure 1:**
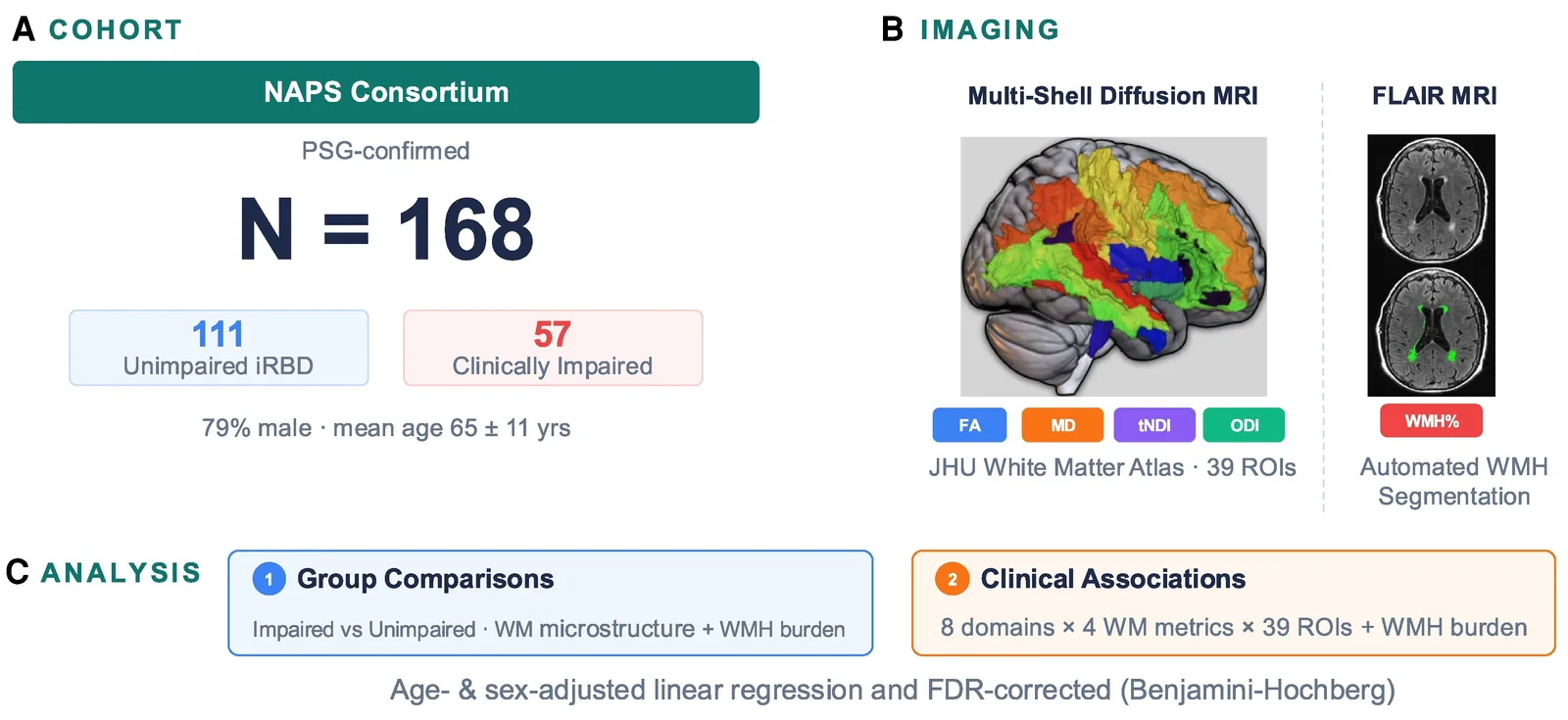
**Study overview. (A)** The study sample comprised 168 participants with polysomnography-confirmed isolated REM sleep behaviour disorder (iRBD) enrolled in the North American Prodromal Synucleinopathy (NAPS) Consortium. **(B)** Multi-shell diffusion MRI was acquired on 3T Siemens PRISMA scanners and processed to derive DTI and NODDI metrics across 39 white matter regions of interest from a modified JHU white matter atlas. WMH burden was quantified from 3D FLAIR images using automated segmentation. **(C)** Aim 1 compared white matter microstructure and WMH burden between unimpaired iRBD and clinically impaired groups. Aim 2 examined associations between white matter metrics and clinical domains. Abbreviations: DTI, diffusion tensor imaging; FDR, false discovery rate; FLAIR, fluid-attenuated inversion recovery; iRBD, isolated REM sleep behaviour disorder; JHU, Johns Hopkins University; NAPS, North American Prodromal Synucleinopathy; NODDI, neurite orientation dispersion and density imaging; ROI, region of interest; WMH, white matter hyperintensity.

## Materials and methods

### Participants

This study included 168 individuals with polysomnography-confirmed iRBD enrolled in the NAPS Consortium from nine centres across the United States and Canada. Eligible participants were adults aged 18 years or older who met International Classification of Sleep Disorders-3 diagnostic criteria for RBD^27^ and were free of clinical parkinsonism and dementia at enrolment, as determined by comprehensive neurological examination performed by movement disorders specialists and neuropsychological assessment. The diagnosis of isolated RBD refers to the absence of neurodegenerative disease at the time of assessment, the presence of comorbid sleep conditions such as obstructive sleep apnoea, periodic limb movement disorder or parasomnias did not preclude iRBD diagnosis, as these conditions are commonly observed in iRBD patients and do not alter the core diagnostic criteria. Given the longitudinal design of our study, participants underwent structured clinical assessments at follow-up visits, and clinical status for the present analysis was defined at the visit corresponding to the diffusion MRI scan. A subset of the sample developed MCI or phenoconverted to an overt synucleinopathy over the course of follow-up. Fifty-seven participants (33.9%) had clinical impairment (mild cognitive impairment, Parkinson’s disease, DLB or MSA) at the time of their diffusion MRI scan. MCI was ascertained through a consensus process involving independent cognitive diagnoses by the site neurologist and neuropsychologist, with the neuropsychological assessment based on demographically adjusted z-scores from the test battery. MCI generally required two or more abnormal scores (z < −1.5) in one or more cognitive domains, followed by consensus review at each visit, as previously described.^28^ The remaining 111 participants (66.1%) were classified as clinically unimpaired. This study was performed in accordance with the Declaration of Helsinki and approved by the Institutional Review Boards at each enrolment site. All participants provided written informed consent prior to participation.

### Clinical and motor assessments

Demographic information and clinical variables were collected for all participants. Cognitive assessment included the Montreal Cognitive Assessment (MoCA).^29,30^ Working memory was evaluated using the digit span forward subtest from the Wechsler Adult Intelligence Scale, Fourth Edition (WAIS-IV), recording the number of correct trials.^31^ Motor function was assessed using the Movement Disorder Society Unified Parkinson’s Disease Rating Scale Part III (MDS-UPDRS-III). Manual dexterity was measured using the Purdue Pegboard test, recording the sum of right hand, left hand and bimanual trials.^32^ Autonomic dysfunction was quantified using the Scales for Outcomes in Parkinson’s Disease-Autonomic (SCOPA-AUT).^33^ Olfactory function was evaluated using the 12-item Brief Smell Identification Test (BSIT).^34^ Prodromal synucleinopathy severity was assessed using the PSRS, a composite measure integrating motor, cognitive, autonomic and sleep features, with higher scores reflecting greater prodromal disease burden.^26^ Daytime sleepiness was measured using the Epworth Sleepiness Scale (ESS), a validated questionnaire that assesses the likelihood of dozing in eight different situations.^35^

### MRI acquisition

All high-resolution structural images were acquired using a 3D magnetization-prepared rapid gradient echo (MPRAGE) sequence on 3T Siemens PRISMA scanners (Siemens Healthcare, Erlangen, Germany), with 1.0 mm isotropic voxels. The field of view was 256 × 256 × 256 mm, and imaging parameters were: repetition time (TR) = 2300 ms, echo time (TE) = 2.98 ms and inversion time (TI) = 900 ms, with a flip angle of 9°. Parallel imaging acceleration factor was 2. Diffusion MRI was acquired with a spin echo single shot Echo Planar Imaging (EPI) sequence at 3T, on Siemens scanners (Siemens Healthcare, Erlangen, Germany), with 2.0 mm isotropic voxels. The field of view was 232 × 232 × 162 mm, with TE of 71 ms and TR of 3400 ms. Each scan consisted of 13 b = 0, 6 b = 500, 48 b = 1000 and 60 b = 2000 s/mm^2^ volumes, with diffusion gradients in each shell evenly distributed using an electrostatic repulsion scheme over whole spheres.^36^ Sagittal 3D FLAIR images were acquired on 3T Siemens scanners (Siemens Healthcare, Erlangen, Germany) using a magnetization-prepared sequence with 1.0 × 1.0 × 1.2 mm^3^ voxel resolution. Imaging parameters were TR = 4800 ms, TE = 441 ms and TI = 1550 ms. All received DICOM files across the participating sites were routed through an in-house automated protocol checking system. Briefly, protocol checks validated several scan parameters, including voxel size, TE and TR, against expected values. Scans passing protocol checks underwent manual image quality control by a team of trained analysts at the Mayo Clinic, who evaluated raw image quality (e.g. head coverage, motion artefacts) and downstream processing success (e.g. segmentation and atlas registration). Images could be excluded at either stage of quality assurance.

### Image processing

#### Diffusion tensor imaging and neurite orientation dispersion and density imaging

The overall diffusion MRI processing workflow is illustrated in Supplementary Fig. 1. Head motion and eddy current distortion were corrected using FSL's eddy program.^37^ A denoized image and noise map were calculated using dwidenoise.^38^ We applied ringing correction^39^ and removed the Rician noise bias using the noise image from the denoising step. A total intracranial volume (TIV) mask was then made from the corrected data and used to skull strip it.^40^ Diffusion tensors were estimated using non-linear least squares fitting to calculate FA and MD maps using the dipy software package.^41^ For NODDI analysis, we calculated NDI, ODI and ISOVF using the AMICO implementation.^42^ To avoid amplifying noise in voxels at the interface between CSF and brain tissue, we calculated tNDI by multiplying NDI by (1-ISOVF).^19^ For regional analysis, we used ANTS^43^ to non-linearly register a modified JHU ‘Eve’ WM atlas^44^ to each subject (FA to FA), effectively correcting for susceptibility distortion by making the atlas conform to the distortion [see Supplementary Table 1 for full region of interest (ROI) labels]. The atlas was modified by fusing the left and right portions of regions crossing the midplane, namely the corpus callosum, pons and fornix. Voxels with MD > 2 × 10^−3^ or <7 × 10^−5^ mm^2^/s were excluded as mostly CSF or air, respectively, and the median FA, MD, tNDI and ODI values were calculated in each ROI. The cuneus, precuneus, fusiform and lingual WM regions were excluded since they were too small for reliable registration.

#### White matter hyperintensities

To quantify WMH burden, we applied a fully automated segmentation algorithm to the MPRAGE and T_2_-weighted FLAIR sequences.^45^ Briefly, WMH voxels are delineated according to their signal intensity and spatial location relative to adjacent grey and white matter. Misclassifications were excluded using a white matter mask generated from SPM12 segmentation of the MPRAGE.^46^ Subsequently, WMH volume was expressed as a percentage of TIV (derived from SPM segmentation).

### Statistical analysis

Demographic and clinical characteristics were summarized using means and standard deviations for continuous variables and frequencies with percentages for categorical variables. For continuous variables, t-tests were done and for categorical variables, chi-square tests were used. WMH volume was divided by TIV (expressed as a percentage) and log-transformed prior to all analyses to address right skew. First, white matter microstructure and then WMH burden were compared between impaired and unimpaired iRBD participants using linear regression models adjusted for age and sex. Sensitivity analyses repeated microstructure comparisons with additional adjustment for WMH burden to assess whether group differences were independent of cerebrovascular burden. Second, we examined associations between white matter microstructure and eight clinical measures: MoCA total score, digit span forward score, Movement Disorder Society Unified Parkinson’s Disease Rating Scale Part III (MDS-UPDRS III) total score, Purdue Pegboard sum score, SCOPA-AUT total score, BSIT score, PSRS total score and ESS total score. For each clinical variable, separate linear regression models were fitted with the clinical measure as the predictor and the diffusion metric as the outcome, adjusting for age and sex. FDR-corrected *P*-values (*q*-values) were computed using the Benjamini–Hochberg procedure across the 39 regions of interest within each family of tests. For group comparisons, FDR correction was applied separately for each diffusion metric. For clinical association analyses, we applied FDR correction separately for each clinical predictor-and-diffusion-metric combination. Associations with *q* < 0.05 were considered statistically significant. Multiple comparisons from the DTI and NODDI analyses were controlled using FDR correction with the Benjamini–Hochberg procedure across the 39 regions of interest with FDR *q* < 0.05 considered statistically significant. All statistical analyses were performed in R (version 4.5.1).

## Results

### Participant characteristics

The study sample comprised 168 iRBD patients (79% male; mean age 65.4 ± 10.6 years; Table 1). Clinical impairment was present in 57 patients (34%). Compared to 111 unimpaired patients (66%), impaired patients were significantly older (69.2 versus 63.4 years, *P* < 0.001) and demonstrated worse performance across multiple domains. Cognitive deficits in the impaired group included lower global cognition (MoCA: 25.1 versus 26.8, *P* = 0.001) and reduced working memory (digit span forward: 7.6 versus 8.8, *P* < 0.001). The impaired group also showed greater motor dysfunction (MDS-UPDRS Part III: 12.1 versus 5.6, *P* < 0.001), greater autonomic dysfunction (SCOPA-AUT: 15.4 versus 12.1, *P* = 0.003), greater clinical severity (PSRS: 10.8 versus 6.3, *P* < 0.001), olfactory impairment (BSIT: 6.5 versus 8.1, *P* = 0.002), reduced fine motor dexterity (Purdue Pegboard: 19.7 versus 23.5, *P* < 0.001). However, daytime sleepiness did not differ between groups (ESS: 6.8 versus 6.2, *P* = 0.39).

**Table 1:** Demographic and clinical characteristics

	Overall *n* = 168	Unimpaired iRBD *n* = 111	Impaired iRBD *n* = 57	Test statistic	*P*-value
Age, yrs	65.4 (10.6)	63.4 (11.0)	69.2 (8.8)	t(166) = −3.43	<0.001
Males, no. (%)	132 (79%)	83 (75%)	49 (86%)	χ^2^(1) = 2.80	0.094
Education, yrs	16.4 (2.6)	16.4 (2.7)	16.3 (2.5)	t(166) = 0.31	0.75
MoCA	26.2 (3.2)	26.8 (2.9)	25.1 (3.5)	t(166) = 3.32	0.001
Digit span forward	8.4 (2.2)	8.8 (2.2)	7.6 (2.0)	t(166) = 3.61	<0.001
MDS-UPDRS III	7.8 (10.3)	5.6 (8.0)	12.1 (12.8)	t(165) = −4.03	<0.001
SCOPA-AUT	13.2 (6.9)	12.1 (6.8)	15.4 (6.6)	t(166) = −2.97	0.003
PSRS	7.9 (4.8)	6.3 (4.0)	10.8 (4.9)	t(164) = −6.34	<0.001
ESS	6.4 (4.4)	6.2 (4.5)	6.8 (4.1)	t(165) = −0.86	0.39
BSIT	7.5 (3.1)	8.1 (3.2)	6.5 (2.7)	t(164) = 3.13	0.002
Purdue Pegboard	22.2 (4.8)	23.5 (4.2)	19.7 (4.8)	t(165) = 5.22	<0.001

Values represent mean (standard deviation) and two-sample *t*-test for continuous variables and count (percentage) and chi-squared test for categorical variables. MoCA ranges from 0–30, with scores ≥26 considered normal. The MDS-UPDRS-III ranges from 0–132, with higher scores indicating more severe motor symptoms. Digit span forward ranges from 0–14, with higher scores indicating better working memory performance. The BSIT ranges from 0–12, with higher scores representing better olfactory function. The PSRS ranges from 0–25, with higher scores indicating greater prodromal disease burden. The SCOPA-AUT ranges from 0–69, with higher scores indicating greater autonomic dysfunction. The Purdue Pegboard Test total score measures manual dexterity and coordination, with higher scores indicating better performance. The ESS ranges from 0–24, with scores >10 suggesting excessive daytime sleepiness.

Abbreviations: BSIT, Brief Smell Identification Test; ESS, Epworth Sleepiness Scale; iRBD, isolated REM sleep behaviour disorder; MDS-UPDRS III, Movement Disorder Society Unified Parkinson's Disease Rating Scale Part III; MoCA, Montreal Cognitive Assessment; PSRS, Prodromal Synucleinopathy Rating Scale; SCOPA-AUT, Scales for Outcomes in Parkinson's Disease–Autonomic.

### Group comparisons of white matter microstructure and hyperintensities

#### iRBD with clinical impairment versus unimpaired iRBD

Group comparisons between clinically impaired and unimpaired iRBD participants, adjusted for age and sex, revealed limited white matter differences after FDR correction (Figs 2 and 3). Impaired participants demonstrated lower FA in the superior fronto-occipital fasciculus (β = −0.025, 95% CI [−0.038, −0.011], FDR *q* = 0.010) and inferior occipital white matter (β = −0.015, 95% CI [−0.024, −0.007], FDR *q* = 0.010), alongside higher ODI in inferior occipital white matter (β = 0.010, 95% CI [0.004, 0.016], FDR *q* = 0.021). Impaired patients showed significantly higher log-transformed WMH burden (β = 0.242, 95% CI [0.021, 0.462], *P* = 0.032), adjusting for age and sex (Fig. 4). After adjusting for log-transformed WMH burden, both FA reductions were no longer significant, with only elevated ODI in inferior occipital white matter remaining independent (β = 0.012, 95% CI [0.007, 0.018], FDR *q* = 0.002, Supplementary Fig. 2). Raw value distributions for each diffusion metric are shown in Supplementary Fig. 3.

**Figure 2:**
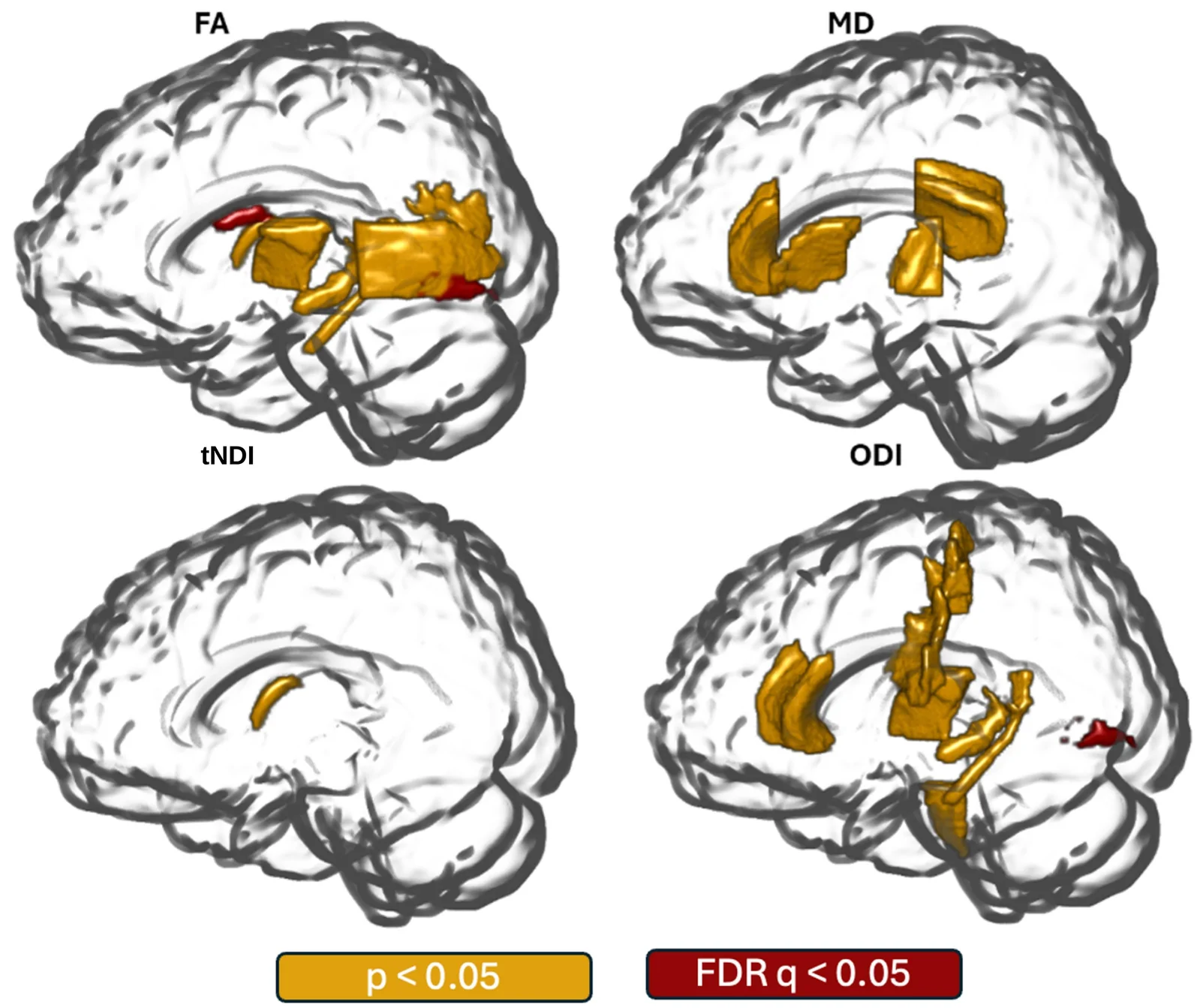
**White matter group differences between impaired participants and unimpaired iRBD participants.** Glass brain maps display white matter regions showing nominal group differences (*P* < 0.05) and those surviving false discovery rate correction (FDR *q* < 0.05) between impaired or phenoconverted participants (*n* = 57) and clinically unimpaired iRBD participants (*n* = 111), based on linear regression models adjusted for age and sex. Abbreviations: FA, fractional anisotropy; FDR, false discovery rate; iRBD, isolated REM sleep behaviour disorder; MD, mean diffusivity; ODI, orientation dispersion index; tNDI, tissue-weighted neurite density index.

**Figure 3:**
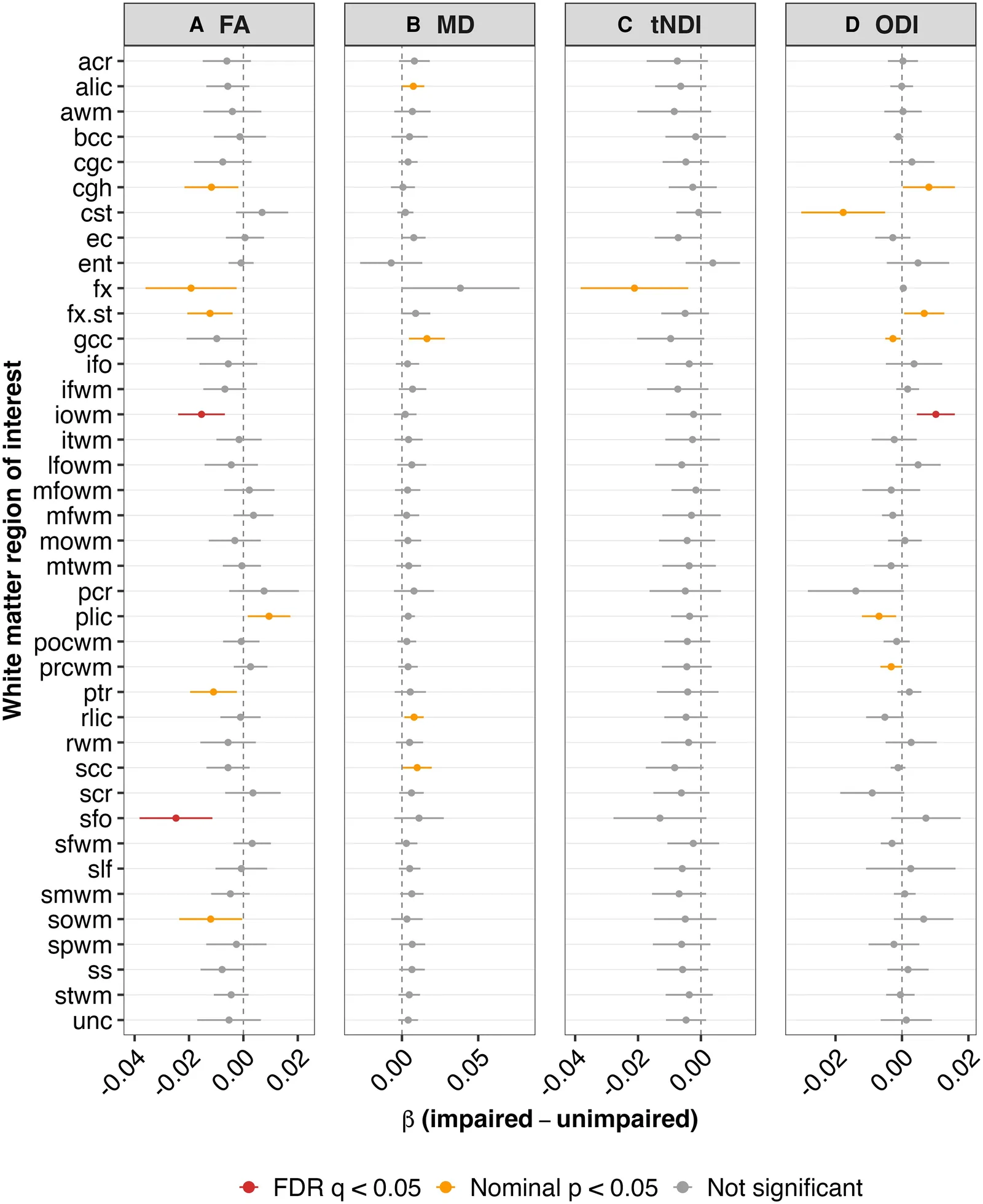
**White matter microstructural differences between impaired participants and unimpaired iRBD participants**. Each subfigure shows the β coefficients (95% CI) from linear regression models that were adjusted for age and sex, comparing clinically unimpaired iRBD participants (*n* = 111) and clinically impaired or phenoconverted participants (*n* = 57). **A**, FA; **B**, MD; **C**, tNDI; **D**, ODI. The point colours represent the level of statistical significance: red = FDR *q* < 0.05; orange = uncorrected *P* < 0.05; grey = not significant. Raw value distributions for each metric are shown in boxplots provided in Supplementary Fig. 3. Abbreviations: CI, confidence interval; FA, fractional anisotropy; FDR, false discovery rate; iRBD, isolated REM sleep behaviour disorder; MD, mean diffusivity; ODI, orientation dispersion index; tNDI, tissue-weighted neurite density index. See Supplementary Table 1 for a complete list of region-of-interest abbreviations.

**Figure 4:**
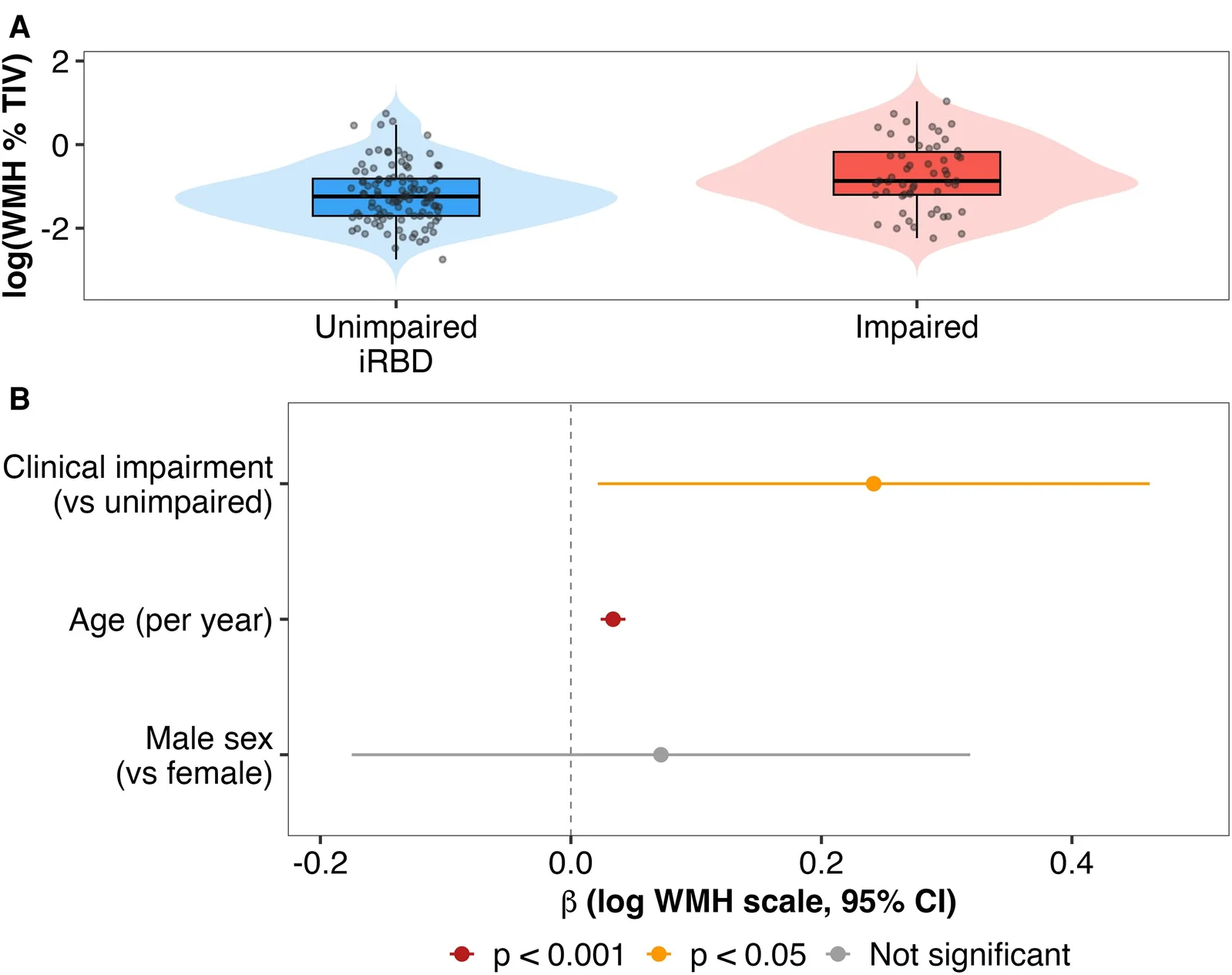
**Comparisons of white matter hyperintensity burden between the impaired group and unimpaired iRBD participants.** (**A**) Distributions of log-transformed white matter hyperintensity burden in clinically unimpaired iRBD (*n* = 108) and clinically impaired (*n* = 54) participants. Six participants had missing WMH data and were excluded from this analysis. Of the six participants with missing WMH data, three were unimpaired and three were impaired. Each datapoint represents an individual participant. (**B**) Forest plot showing β coefficients (95% CI) from a linear regression model examining predictors of log-transformed WMH burden, including clinical impairment status, age and sex. Clinical impairment (versus unimpaired): β = 0.242 [0.021, 0.462], *P* = 0.032. Age (per year): β = 0.034 [0.024, 0.044], *P* < 0.001. Male sex (versus female): β = 0.072 [−0.175, 0.319], *P* = 0.566. Abbreviations: CI, confidence interval; iRBD, isolated REM sleep behaviour disorder; TIV, total intracranial volume; WMH, white matter hyperintensity.

#### Associations of clinical variables with white matter microstructure and hyperintensity burden

Clinical associations with white matter microstructure are summarized in Fig. 5. Working memory, indexed with digit forward span, demonstrated the most extensive microstructural associations, with 65 FDR-significant findings across FA, MD and tNDI metrics. Poorer digit span forward performance was associated with lower FA in 5 regions (external capsule, inferior fronto-occipital fasciculus, posterior corona radiata, superior longitudinal fasciculus and sagittal stratum), higher MD in 31 ROIs and lower tNDI in 29 regions. Autonomic dysfunction demonstrated widespread microstructural associations, with 41 FDR-significant findings across FA, MD and tNDI. Greater SCOPA-AUT symptom severity was associated with lower FA in 8 regions, higher MD in 15 regions and lower tNDI in 18 regions. Significant associations involved bilateral association tracts, commissural pathways and frontal and temporal white matter and the fornix/stria terminalis. Other clinical domains showed minimal or no significant microstructural associations. Prodromal disease severity (PSRS) demonstrated two FDR-significant associations (lower FA in the superior fronto-occipital fasciculus and fornix), while olfactory function (BSIT) showed one association (higher tNDI in the fornix/stria terminalis). Global cognition (MoCA), motor severity (MDS-UPDRS III), manual dexterity (Purdue Pegboard) and daytime sleepiness (ESS) showed no associations with any of the DTI or NODDI parameters (all FDR *q* > 0.05).

**Figure 5:**
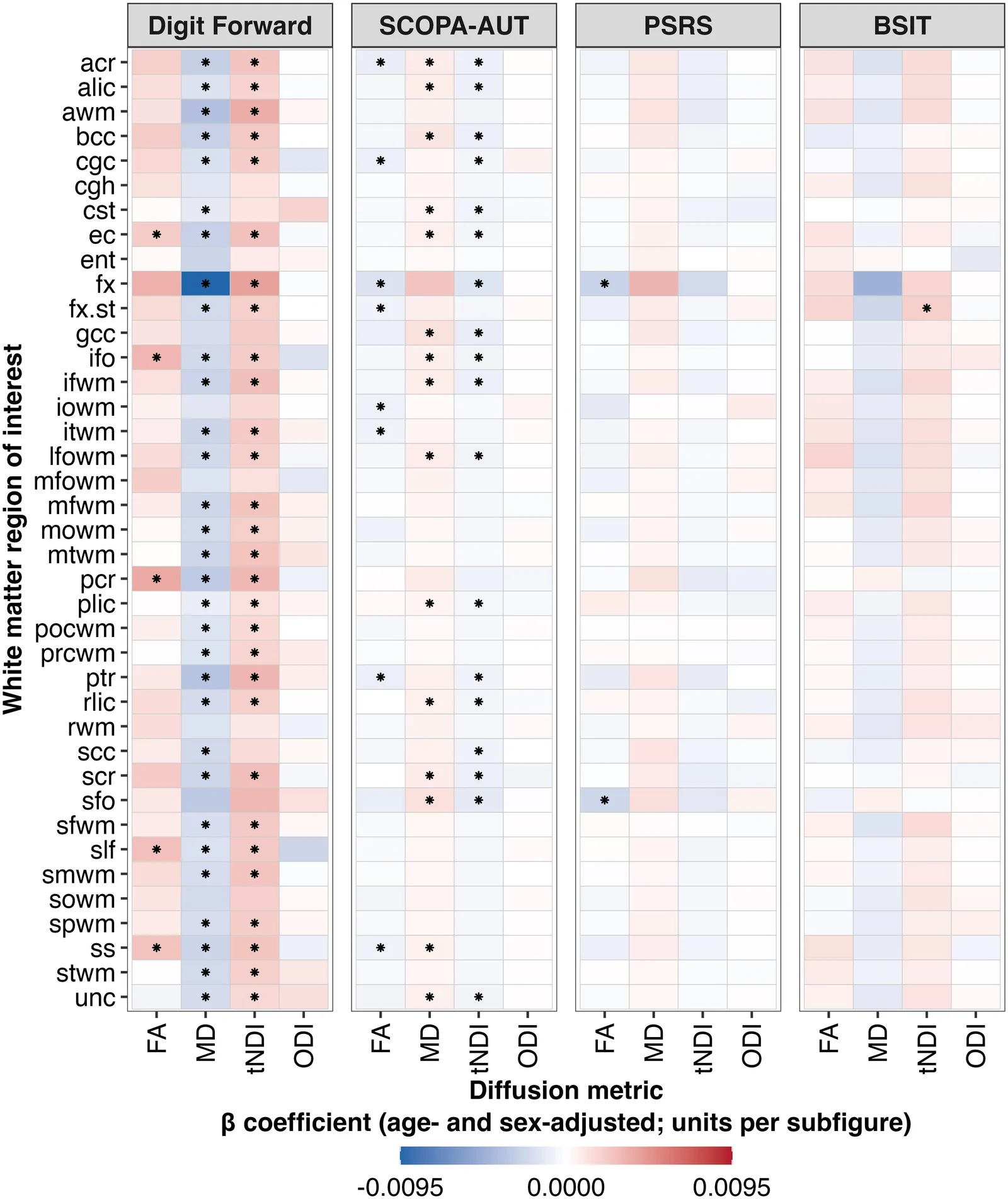
**Clinical associations with white matter microstructure.** Heatmaps show β coefficients from age- and sex-adjusted linear regression models. Analyses were based on *N* = 168 for Digit Forward and SCOPA-AUT and *N* = 166 for PSRS and BSIT. Asterisks indicate FDR *q* < 0.05. Blue = negative association; red = positive association. Abbreviations: BSIT, Brief Smell Identification Test; FA, fractional anisotropy; FDR, false discovery rate; MD, mean diffusivity; ODI, orientation dispersion index; PSRS, Prodromal Synucleinopathy Rating Scale; SCOPA-AUT, Scales for Outcomes in Parkinson's Disease–Autonomic; tNDI, tissue-weighted neurite density index. See Supplementary Table 1 for a complete list of region-of-interest abbreviations.

WMH burden demonstrated broad associations with clinical variables (Fig. 6). In age- and sex-adjusted linear regression models, higher log-transformed WMH burden was significantly associated with greater prodromal disease severity (PSRS: β = 0.033, 95% CI [0.011, 0.056], *P* = 0.004), poorer global cognition (MoCA: β = −0.042, 95% CI [−0.074, −0.010], *P* = 0.010), poorer working memory performance (digit span forward: β = −0.052, 95% CI [−0.098, −0.006], *P* = 0.026) and reduced manual dexterity (Purdue Pegboard: β = −0.027, 95% CI [−0.051, −0.002], *P* = 0.036). WMH burden was not significantly associated with autonomic dysfunction (SCOPA-AUT), daytime sleepiness (ESS), motor severity (MDS-UPDRS III) or olfactory function (BSIT).

**Figure 6:**
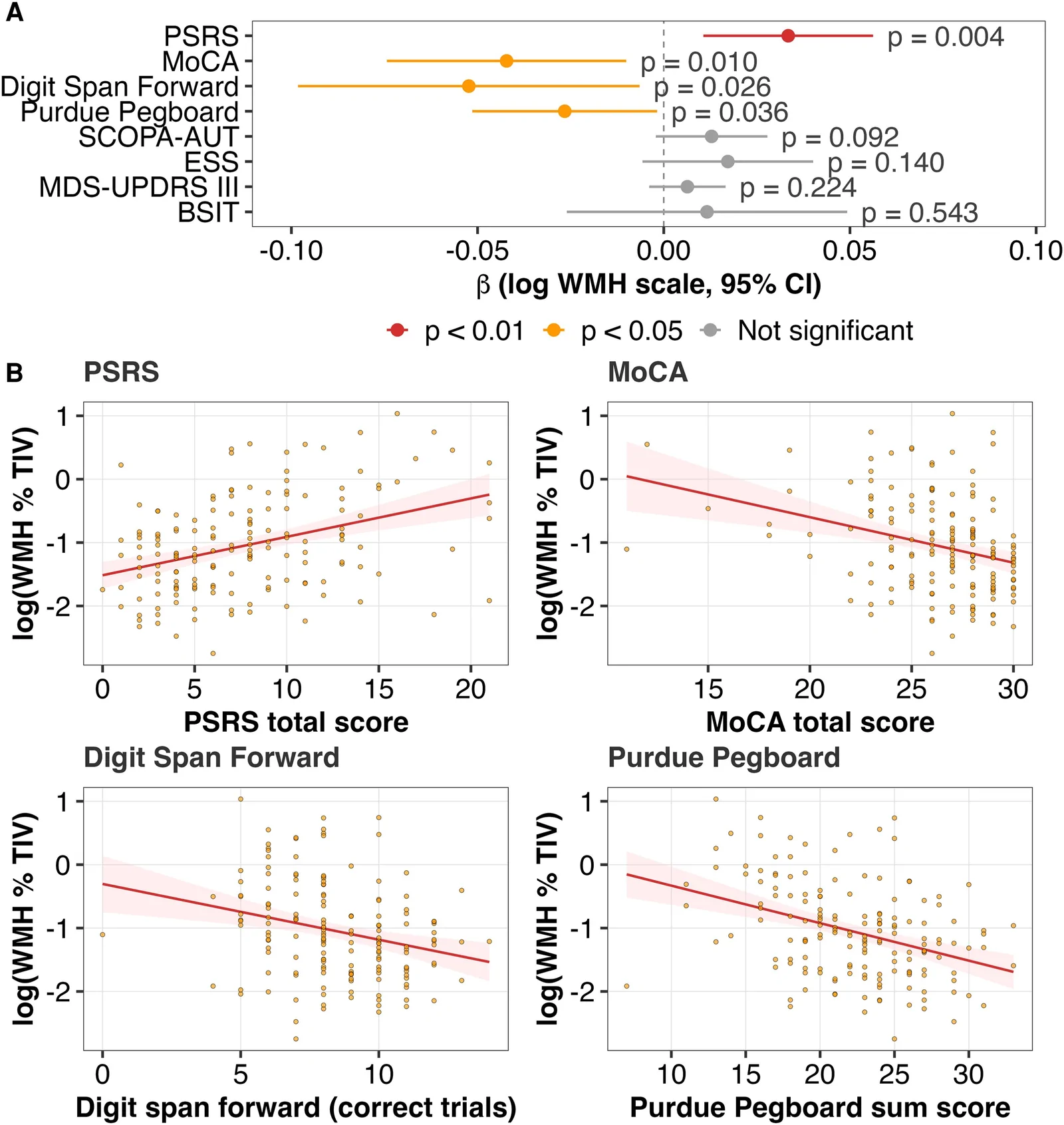
**Associations of white matter hyperintensity burden with clinical and cognitive function.** (**A**) Forest plot showing β coefficients (95% CI) from age- and sex-adjusted linear regression models of log-transformed WMH burden (% TIV) across the clinical domains (*N* = 162 for MoCA, SCOPA-AUT and Digit Span Forward; *N* = 161 for MDS-UPDRS III, PSRS, ESS and Purdue Pegboard; *N* = 160 for BSIT). The point colours indicate significance: red = *P* < 0.01; orange = *P* < 0.05; grey = not significant. (**B**) Scatter plots with regression lines (shaded 95% CI) for the four statistically significant associations; each datapoint represents a participant. Abbreviations: BSIT, Brief Smell Identification Test; CI, confidence interval; ESS, Epworth Sleepiness Scale; MDS-UPDRS III, Movement Disorder Society Unified Parkinson's Disease Rating Scale Part III; MoCA, Montreal Cognitive Assessment; PSRS, Prodromal Synucleinopathy Rating Scale; SCOPA-AUT, Scales for Outcomes in Parkinson's Disease–Autonomic; TIV, total intracranial volume; WMH, white matter hyperintensity.

## Discussion

### Main findings

In this multi-site study of 168 iRBD participants, we combined multi-shell diffusion and FLAIR imaging to characterize the extent of microstructural and cerebrovascular white matter injury in prodromal α-synucleinopathy and delineate their associations with clinical impairment and domain-specific functional deficits. Three key findings emerged. First, clinical impairment was associated with elevated WMH burden but only circumscribed microstructural differences on diffusion MRI. Second, microstructural injury on DTI and NODDI showed domain-selective relationships, most prominently with working memory and autonomic dysfunction, whereas WMH burden showed broad associations across multiple clinical domains, most notably with overall prodromal disease severity as indexed by the PSRS, a composite measure capturing cognitive, motor, autonomic, behavioural, sleep and sensory features. Third, after adjustment for WMH burden, microstructural differences between impaired and unimpaired participants were attenuated, with only elevated ODI in inferior occipital white matter retaining an independent association.

### Minimal white matter microstructural differences and elevated WMH with clinical impairment

Despite substantial clinical differences between impaired and unimpaired iRBD participants (Table 1), there were remarkably limited white matter microstructural differences seen on DTI and NODDI after age and sex adjustment, confined to lower FA in the superior fronto-occipital fasciculus and inferior occipital white matter, and elevated ODI in inferior occipital white matter. Moreover, after adjustment for WMH burden, both FA reductions were no longer significant, with only elevated ODI in inferior occipital white matter retaining independence. This dissociation suggests that FA white matter differences in clinically impaired iRBD likely reflect shared cerebrovascular burden rather than primary neurodegenerative axonal injury alone. In contrast, elevated ODI in the white matter could be taken to reflect increased angular dispersion of axonal fibres, consistent with early neurodegenerative disorganization of otherwise coherent fibre bundles.^47^ The persistence of the ODI signal in inferior occipital white matter after WMH adjustment points to an occipital WM vulnerability that is characteristic of the Lewy body disease continuum. Indeed, converging evidence from various neuroimaging modalities supports early posterior cortical involvement in prodromal Lewy body disease. First, at the macrostructural level, occipital and posterior parieto-temporal atrophy has been identified as an early signature of prodromal DLB.^48^ Second, occipital hypometabolism on FDG-PET has been proposed as a specific risk factor for phenoconversion to Parkinson’s disease in iRBD,^49^ and is also a characteristic feature of DLB, with hypoperfusion in posterior parieto-occipital cortices associated with worse global clinical severity.^50^ In keeping with this, occipital hypometabolism has also been observed in CSF α-synuclein seed amplification assay-positive individuals within the ADNI cohort, even in the absence of overt clinical Lewy body disease,^51^ suggesting that posterior metabolic vulnerability may emerge early along the synucleinopathy spectrum. Thirdly, focal ODI reductions in occipital grey matter have been observed across the DLB spectrum,^52^ reflecting dendritic loss and reduced synaptic complexity in a region of established posterior vulnerability.^16^ Taken together, the elevated ODI in occipital white matter observed in our clinically impaired iRBD patients may constitute an early white matter substrate of the well-established posterior cortical vulnerability in Lewy body disease, although whether elevated occipital ODI represents an early marker of posterior-predominant Lewy body pathology with prognostic utility for phenoconversion subtype remains a hypothesis to be tested in future longitudinal studies. Interestingly, there is evidence from animal models suggesting that REM sleep supports activity-dependent synaptic pruning,^53^ raising the possibility that chronic REM sleep disruption in iRBD could similarly impair this process, although this remains speculative given the lack of histopathological validation of NODDI metrics.

In contrast to the minimal white matter tract findings, impaired participants showed significantly elevated WMH burden compared to unimpaired participants, suggesting that in prodromal synucleinopathy, cerebrovascular burden is more closely associated with clinical impairment than regional white matter microstructural differences. These findings support a dual-hit model in which α-synuclein-related neurodegeneration and cerebrovascular pathology interact to lower the threshold for clinical expression. Consistent with this, neuropathological data indicate that cerebrovascular pathology inversely correlates with the burden of Lewy pathology required to produce cognitive impairment, implying that less neurodegenerative injury is needed to reach clinical thresholds in the presence of vascular co-pathology.^21,54^

Importantly, our findings are in keeping with a substantial body of evidence linking WMH burden to clinical dysfunction across established Parkinson’s disease and DLB. In Parkinson’s disease, WMH burden increases across the cognitive spectrum and is associated with cognitive decline.^23,55^ Longitudinal community-based studies further show that elevated WMH volume is associated with increased risks of incident parkinsonism,^56^ while meta-analytic evidence demonstrates associations with worse cognitive and motor outcomes across the Parkinson’s disease spectrum.^57^ In DLB, WMH burden was particularly pronounced in posterior (parieto-occipital) regions,^24^ and also closely related to core clinical features and neurodegeneration.^24,58-60^ Extending these observations to the prodromal stage, a recent retrospective study of 372 iRBD patients from the Mayo Clinic, demonstrated that vascular comorbidity was associated with higher phenoconversion rates to overt synucleinopathy, even after accounting for age, sex and antidepressant use.^6^ Taken together, our findings extend prior work by demonstrating, for the first time in a large multi-site iRBD cohort, that greater cerebrovascular lesion load measured with WMH is a significant contributor to clinical impairment in prodromal synucleinopathy.

### Clinical associations of WMH and white matter microstructure

Greater WMH burden was associated with worse performance across multiple clinical domains, including global cognition, working memory and manual dexterity, with the strongest association observed for the PSRS. Moreover, meta-analytic evidence indicates that greater WMH burden is correlated with poorer global cognitive and motor performance in Parkinson’s disease,^57^ and our findings suggest that similar relationships could be salient even at the prodromal iRBD stage. Moreover, the associations with global cognition and working memory are consistent with prior work implicating WMH in frontal and subcortical network disruption, with lesion probability mapping in Parkinson’s disease specifically linking WMH to executive function and attention/working memory domains through involvement of frontal and subcortical pathways.^61^ The mechanisms through which small vessel disease exerts such broad clinical effects are increasingly understood in terms of structural network disruption, whereby WMH-related disruptions of cortical-subcortical and cortico-cortical connections impede brain networks underlying domains such as executive function and processing speed.^62^ Our findings may have clinical implications too. Unlike α-synuclein pathology, cerebrovascular burden is substantially modifiable. Hypertension treatment, for example, has been shown to reduce WMH progression and slow the accumulation of white matter disease in randomized controlled trials,^63^ raising the possibility that more aggressive vascular risk factor management could attenuate the cerebrovascular contribution to clinical progression and potentially delay phenoconversion in patients with iRBD.

In contrast to the broad clinical associations observed for WMH, diffusion MRI microstructural metrics demonstrated a more selective pattern of associations, most prominently with working memory (digit span forward) and autonomic dysfunction across widespread white matter tracts. Recent DTI studies in iRBD have reported associations between white matter integrity and clinical outcomes, with diffusion metrics showing promise as prodromal biomarkers of phenoconversion,^64^ and prior work in individuals at risk for Parkinson’s disease has implicated frontoparietal white matter integrity to executive function and cognitive flexibility.^65^ As such, the present findings extend this literature by demonstrating that multi-shell diffusion metrics, particularly tNDI, capture working memory-related microstructural variance across a broader topographic distribution than has previously been reported, encompassing association, commissural and projection tracts. The coupling between white matter microstructural integrity and working memory performance is well established, with axonal volume and myelination in specific white matter tracts correlating with performance on working memory tasks across cohorts of neurological populations.^66^

Autonomic dysfunction is a core and early feature of α-synucleinopathies that typically precede motor symptom onset across Parkinson’s disease, DLB and MSA, arising from both peripheral and central nervous system involvement.^67^ In our study, the microstructural correlates of autonomic dysfunction were similarly widespread, spanning bilateral association tracts, commissural pathways and frontal and temporal white matter, with particularly robust effects in the fornix and stria terminalis. To our knowledge, this is the first study to directly examine associations between white matter microstructure and autonomic dysfunction as indexed by SCOPA-AUT in iRBD. Interestingly, the directionality of these associations stands in stark contrast to prior DTI connectomic findings in early drug-naïve Parkinson’s disease from the Parkinson’s Progression Markers Initiative, which reported positive associations between SCOPA-AUT scores and white matter connectivity interpreted as reflecting neural compensation in early Parkinson’s disease.^68^ However, differences in disease stage, diffusion methodology (DTI and NODDI versus spin distribution function) and analytic approach preclude direct comparisons.

### Limitations and future directions

Several limitations are worth considering. First, the cross-sectional design precludes inference about the temporal sequence of white matter changes relative to clinical progression. Whether microstructural and cerebrovascular injury precede, accompany in parallel or follow clinical decline cannot be determined from our design. Longitudinal analyses within the NAPS Consortium, where participants undergo repeat imaging at two-to-three-year intervals, will be essential to address this question. Second, our within-iRBD design did not include a healthy control group, precluding direct assessment of whether the microstructural and WMH patterns observed reflect disease-specific processes or age-related changes. Nevertheless, our study was designed to characterize within-group heterogeneity in white matter injury and its relationship to clinical function across the impairment spectrum in iRBD.^26^ Third, WMH quantification was performed using automated segmentation without regional parcellation, constraining our ability to determine whether the topography of WMH (i.e. lobar WMHs versus periventricular versus deep WMH) could differentially relate to the observed clinical associations, as demonstrated by distinct spatial associations between WMH and cognition in Parkinson’s disease.^69^ Future work looking at lobar or periventricular/deep white matter WMHs could enable more direct comparison with the present regional diffusion MRI findings. On a related note, WMH on FLAIR MRI generally reflects a non-specific increase in tissue water that may arise from causes other than cerebrovascular injury, such as demyelination and extracellular fluid accumulation, and should not be interpreted exclusively as a specific marker of small vessel disease. Fourth, the impaired group comprised iRBD participants with mild cognitive impairment, dementia and phenoconversion to clinically defined synucleinopathies including Parkinson’s disease, DLB and MSA. This heterogeneity, while reflecting the real-world clinical scenario of prodromal synucleinopathy, may have diluted group-level microstructural differences that could be more pronounced when stratified by specific phenotypic groups. Fifth, our iRBD sample was predominantly male, consistent with the known demographics of clinically diagnosed iRBD but limiting generalizability to female participants. Future studies are warranted to explicitly examine sex as a biological variable, including the influence of sex-specific life-course exposures on white matter integrity and vascular burden in iRBD.^70^ Sixth, comorbid sleep conditions such as obstructive sleep apnoea and periodic limb movement disorder were not systematically characterized or adjusted for in our analyses. Both conditions may have independent associations with white matter injury via mechanisms such as chronic intermittent hypoxia and vascular mechanisms in the case of obstructive sleep apnoea, and sleep fragmentation in the case of periodic limb movement disorder.^71^ Future work incorporating more precise comorbidity data across the NAPS Consortium will be needed to disentangle their contribution from synucleinopathy-related processes. In addition, future studies are needed to disentangle the relative contributions of cerebrovascular injury and α-synuclein-related neurodegeneration to microstructural imaging changes and clinical severity in iRBD. Longitudinal cohorts featuring serial structural imaging, α-synuclein seed amplification assays^51,72^ or emerging PET imaging of α-synuclein^73^ will be needed to determine whether WMH progression could interact synergistically with α-synuclein pathology in shaping the clinical trajectory of iRBD.

## Conclusions

In summary, our findings of elevated WMH burden in clinically impaired iRBD participants, together with its association with global clinical severity (PSRS), support a ‘double-hit’ model in which cerebrovascular lesions interact with early neurodegenerative processes to shape clinical expression in prodromal synucleinopathy. Longitudinal studies incorporating serial diffusion and FLAIR imaging with clinical follow-up will be essential to determine the temporal evolution and prognostic significance of these markers.

## Supplementary Material

fcag414_Supplementary_Data

## Data Availability

The datasets are available from the corresponding author upon reasonable request and with appropriate institutional approvals. In addition, the analysis script used for this study is provided in the Supplementary Material.
