# Supplementary material for: White matter microstructure and hyperintensity burden in isolated REM sleep behaviour disorder
Source: Brain Commun. 2026 Nov 2;8(6):fcag414. doi: 10.1093/braincomms/fcag414 (PMC13764781; doi:10.1093/braincomms/fcag414)
Supplement: Supplementary file 1 [file fcag414_supplementary_data.docx]

# Supplementary material

| **ROI Label** | **Full Name** |
| --- | --- |
| ACR | Anterior corona radiata |
| ALIC | Anterior limb of internal capsule |
| AWM | Anterior white matter |
| BCC | Body of corpus callosum |
| CGC | Cingulum (cingulate gyrus) |
| CGH | Cingulum (hippocampus) |
| CST | Corticospinal tract |
| EC | External capsule |
| ENT | Entorhinal white matter |
| Fx | Fornix |
| Fx_ST | Fornix / stria terminalis |
| GCC | Genu of corpus callosum |
| IFO | Inferior fronto-occipital fasciculus |
| IFWM | Inferior frontal white matter |
| IOWM | Inferior occipital white matter |
| ITWM | Inferior temporal white matter |
| LFOWM | Lateral fronto-orbital white matter |
| MFOWM | Medial fronto-orbital white matter |
| MFWM | Medial frontal white matter |
| MOWM | Medial occipital white matter |
| MTWM | Middle temporal white matter |
| PCR | Posterior corona radiata |
| PLIC | Posterior limb of internal capsule |
| PTR | Posterior thalamic radiation |
| PoCWM | Post-central white matter |
| PrCWM | Pre-central white matter |
| RLIC | Retrolenticular part of internal capsule |
| RWM | Retrosplenial white matter |
| SCC | Splenium of corpus callosum |
| SCR | Superior corona radiata |
| SFO | Superior fronto-occipital fasciculus |
| SFWM | Superior frontal white matter |
| SLF | Superior longitudinal fasciculus |
| SMWM | Superior medial white matter |
| SOWM | Superior occipital white matter |
| SPWM | Superior parietal white matter |
| SS | Sagittal stratum |
| STWM | Superior temporal white matter |
| UNC | Uncinate fasciculus |

**Supplementary Table 1. Full labels of the white matter ROIs in the study.** This table lists the abbreviations used for the DTI and NODDI metrics extracted from white matter regions of interest (ROIs) based on a modified version of the JHU “Eve” white matter atlas. Abbreviations: DTI = Diffusion Tensor Imaging; NODDI = Neurite Orientation Dispersion and Density Imaging; ROI = Region of Interest; JHU = Johns Hopkins University.

**Supplementary Figure 1. Diffusion MRI processing and analysis pipeline.** Overview of the preprocessing steps for diffusion MRI data and regional analysis workflow. Abbreviations: FA = fractional anisotropy; MD = mean diffusivity; tNDI = tissue-weighted neurite density index; ODI = orientation dispersion index; ROI = region of interest; NDI = neurite density index; ISOVF = isotropic volume fraction; DTI = diffusion tensor imaging; NODDI = neurite orientation dispersion and density imaging; TIV = total intracranial volume; WM = white matter; ANTs = Advanced Normalization Tools.

**Supplementary Figure 2. White matter microstructural differences between the impaired participants and unimpaired iRBD participants after adjustment for WMH.** Each subfigure shows the β coefficients (95% CI) from linear regression models that were adjusted for age and sex and log-transformed WMH burden, comparing clinically unimpaired iRBD participants (n = 108) and clinically impaired participants (n = 54). (A) FA, (B) MD, (C) tNDI, (D) ODI. The point colors represent the level of statistical significance: red = FDR q < 0.05; orange = uncorrected p < 0.05; grey = not significant. Abbreviations: CI, confidence interval; FA, fractional anisotropy; FDR, false discovery rate; iRBD, isolated REM sleep behavior disorder; MD, mean diffusivity; ODI, orientation dispersion index; tNDI, tissue-weighted neurite density index. See **Supplementary Table 1** for a complete list of region-of-interest abbreviations.

**Supplementary Figure 3. Raw value distributions of white matter microstructural metrics in unimpaired and impaired iRBD participants.** (A) FA, (B) MD, (C) tNDI, (D) ODI, comparing clinically unimpaired iRBD participants (blue, n = 111) and clinically impaired (red, n = 57). Boxes show the median and interquartile range. The x-axis in each panel represents the scale for each metric. FA, tNDI, and ODI are dimensionless (range 0–1), while MD is expressed in ×10⁻³ mm²/s. Abbreviations: FA, fractional anisotropy; iRBD, isolated REM sleep behavior disorder; MD, mean diffusivity; ODI, orientation dispersion index; tNDI, tissue-weighted neurite density index. See Supplementary Table 1 for a complete list of region-of-interest abbreviations.

**Analysis Script**

d <- sas.read("../data/", "analysis_20260220")

di <- subset(d,impaired==1)

dc <- subset(d,impaired==0)

di$ref <- "Impaired"

dc$ref <- "RBD"

d$ref <- "All"

d1 <- rbind(d,di,dc)

d1$ref1 <- factor(d1$ref, levels=c("All","RBD","Impaired"))

pval <- function(P) {

ifelse(P < 0.001,"<0.001",

ifelse(P < 0.095,

formatC(P, digits = 3,format = "f"),

formatC(P, digits = 2,format = "f")))

}

rundate <- format(Sys.Date(), "%Y%m%d")

###############################

\## Report

###############################

options(current.figure.num = 1)

options(current.table.num = 1)

f <- paste0("../output/Updated-Characteristics-Table-",rundate,".rtf")

RTF(f)

pard(f, "Characteristics Table", 'h1')

pard(f, today(), 'h2')

w <- c(1.5,1.35,1.35,1.35,.85)

n <- table(d1$ref1); n

trowd(f, TableX("{\\b } Baseline characteristics table with the mean (SD) listed for the continuous variables and count (%) for the categorical variables."), w=sum(w))

trowd(f, c("", paste(levels(d1$ref1), "\\line n = ",n,sep=""),"P-value"), w=w, bty.cell='=')

P <- pval(t.test(d$age.at.visit ~ d$impaired,var.equal = TRUE )$p.value)

trowd(f, c("Age, yrs", tapply(d1$age.at.visit, d1$ref1, meanSD, digits=c(1,1)),P), w=w)

rm(P)

P <- pval(chisq.test(d$impaired, d$sex, correct=FALSE)$p.value)

trowd(f, c("Males, no. (%)", tapply(d1$sex, d1$ref1, noPct, level=1, digits=0),P), w=w)

rm(P)

P <- pval(t.test(d$educ ~ d$impaired,var.equal = TRUE )$p.value)

trowd(f, c("Education, yrs", tapply(d1$educ, d1$ref1, meanSD, digits=c(1,1)),P), w=w)

rm(P)

P <- pval(t.test(d$mocatots ~ d$impaired,var.equal = TRUE )$p.value)

trowd(f, c("MoCA", tapply(d1$mocatots, d1$ref1, meanSD, digits=c(1,1)),P), w=w)

rm(P)

P <- pval(t.test(d$digforct ~ d$impaired,var.equal = TRUE )$p.value)

trowd(f, c("Dig Forward", tapply(d1$digforct, d1$ref1, meanSD, digits=c(1,1)),P), w=w)

rm(P)

P <- pval(t.test(d$mdsupdrs3.total ~ d$impaired,var.equal = TRUE )$p.value)

trowd(f, c("UPDRS", tapply(d1$mdsupdrs3.total, d1$ref1, meanSD, digits=c(1,1)),P), w=w)

rm(P)

P <- pval(t.test(d$scopa.aut.total ~ d$impaired,var.equal = TRUE )$p.value)

trowd(f, c("SCOPA", tapply(d1$scopa.aut.total, d1$ref1, meanSD, digits=c(1,1)),P), w=w)

rm(P)

P <- pval(t.test(d$psrs.sum.score ~ d$impaired,var.equal = TRUE )$p.value)

trowd(f, c("PSRS", tapply(d1$psrs.sum.score, d1$ref1, meanSD, digits=c(1,1)),P), w=w)

rm(P)

P <- pval(t.test(d$epworth.total ~ d$impaired,var.equal = TRUE )$p.value)

trowd(f, c("Epworth", tapply(d1$epworth.total, d1$ref1, meanSD, digits=c(1,1)),P), w=w)

rm(P)

P <- pval(t.test(d$bsit ~ d$impaired,var.equal = TRUE )$p.value)

trowd(f, c("BSIT", tapply(d1$bsit, d1$ref1, meanSD, digits=c(1,1)),P), w=w)

rm(P)

P <- pval(t.test(d$pp.sum ~ d$impaired,var.equal = TRUE )$p.value)

trowd(f, c("Purdue Sum", tapply(d1$pp.sum, d1$ref1, meanSD, digits=c(1,1)),P), w=w)

rm(P)

trowd(f, c("P-values for differences between groups come from t-test for continuous variables or chi-squared test for categorical."), w=sum(w), bty.cell='=')

RTF.off(f)

\## MODEL 1 where impaired group is the predictor of interest

d$imp.gp <- factor(d$impaired, levels=0:1, labels=c("CU","Imp"))

fit1 <- lm(d$fa.gcc ~ age.at.visit + sex.gp + imp.gp, data=d)

reg2 <- data.frame()

for(i in 1:234){

aa <- d[i+214]

c1 <- paste(colnames(aa))

fit1 <- lm(d[,i+214] ~ age.at.visit + sex.gp + imp.gp, data=d)

rr <- summary(fit1)

t1 <- (rr$coefficients["imp.gpImp","t value"])

p1 <- (rr$coefficients["imp.gpImp","Pr(>|t|)"])

e1 <- round(rr$coefficients["imp.gpImp","Estimate"],digits=6)

se1 <- round(rr$coefficients["imp.gpImp","Std. Error"],digits=6)

l1 <- confint(fit1, "imp.gpImp", level=0.95)[1]

u1 <- confint(fit1, "imp.gpImp", level=0.95)[2]

\##

reg2[i,1] <- colnames(aa[1])

reg2[i,2] <- t1

reg2[i,3] <- p1

reg2[i,4] <- l1

reg2[i,5] <- u1

reg2[i,6] <- e1

rm(fit1,rr,t1,p1,e1,se1,l1,u1)

}

head(reg2)

###############################################################

names(reg2)[1] <- "response"

names(reg2)[2] <- "imp.t"

names(reg2)[3] <- "imp.p"

names(reg2)[4] <- "imp.lcl"

names(reg2)[5] <- "imp.ucl"

names(reg2)[6] <- "imp.e"

reg2$ROI <- toupper(gsub(".", " ", reg2$response, fixed=TRUE))

reg2$ROI2 <- toupper(gsub("fa", " ", reg2$ROI, fixed=TRUE))

reg2$ROI2 <- toupper(gsub("md", " ", reg2$ROI2, fixed=TRUE))

reg2$ROI2 <- toupper(gsub("tndi", " ", reg2$ROI2, fixed=TRUE))

reg2$ROI2 <- toupper(gsub("odi", " ", reg2$ROI2, fixed=TRUE))

reg2$ROI2 <- toupper(gsub("fwf", " ", reg2$ROI2, fixed=TRUE))

reg2$ROI2 <- toupper(gsub("ndi", " ", reg2$ROI2, fixed=TRUE))

\## IMPAIRED regular p-value

reg2$d.imp <- ifelse(reg2$imp.p <0.05,"red","black")

reg2$p.imp <- pval(reg2$imp.p)

\## FDR adjustments

reg2$fdr.imp.p <- p.adjust(reg2$imp.p,method='fdr')

reg2$fdr.d.imp <- ifelse(reg2$fdr.imp.p <0.05,"red","black")

reg2$fdr.p.imp <- pval(reg2$fdr.imp.p)

cfa <- reg2[1:39,]

cmd <- reg2[40:78,]

ctndi <- reg2[79:117,]

codi <- reg2[118:156,]

cfwf <- reg2[157:195,]

cndi <- reg2[196:234,]

\## redo the fdr for each

\## IMPAIRED FDR adjustments

cfa$fdr.imp.p <- p.adjust(cfa$imp.p,method='fdr')

cfa$fdr.d.imp <- ifelse(cfa$fdr.imp.p <0.05,"red","black")

cfa$fdr.p.imp <- pval(cfa$fdr.imp.p)

\## md

cmd$fdr.imp.p <- p.adjust(cmd$imp.p,method='fdr')

cmd$fdr.d.imp <- ifelse(cmd$fdr.imp.p <0.05,"red","black")

cmd$fdr.p.imp <- pval(cmd$fdr.imp.p)

\## tndi

ctndi$fdr.imp.p <- p.adjust(ctndi$imp.p,method='fdr')

ctndi$fdr.d.imp <- ifelse(ctndi$fdr.imp.p <0.05,"red","black")

ctndi$fdr.p.imp <- pval(ctndi$fdr.imp.p)

\## odi

codi$fdr.imp.p <- p.adjust(codi$imp.p,method='fdr')

codi$fdr.d.imp <- ifelse(codi$fdr.imp.p <0.05,"red","black")

codi$fdr.p.imp <- pval(codi$fdr.imp.p)

\## fwf

cfwf$fdr.imp.p <- p.adjust(cfwf$imp.p,method='fdr')

cfwf$fdr.d.imp <- ifelse(cfwf$fdr.imp.p <0.05,"red","black")

cfwf$fdr.p.imp <- pval(cfwf$fdr.imp.p)

\## ndi

cndi$fdr.imp.p <- p.adjust(cndi$imp.p,method='fdr')

cndi$fdr.d.imp <- ifelse(cndi$fdr.imp.p <0.05,"red","black")

cndi$fdr.p.imp <- pval(cndi$fdr.imp.p)

\## model 2 adjusting for age and sex with clinical/cognitive as predictors 3/5/26

reg1 <- data.frame()

for(i in 1:234){

aa <- d[i+214]

c1 <- paste(colnames(aa))

fit1 <- lm(d[,i+214] ~ age.at.visit + sex.gp + mocatots, data=d)

rr <- summary(fit1)

t1 <- (rr$coefficients["mocatots","t value"])

p1 <- (rr$coefficients["mocatots","Pr(>|t|)"])

e1 <- round(rr$coefficients["mocatots","Estimate"],digits=6)

se1 <- round(rr$coefficients["mocatots","Std. Error"],digits=6)

l1 <- confint(fit1, "mocatots", level=0.95)[1]

u1 <- confint(fit1, "mocatots", level=0.95)[2]

\## digforct

fit2 <- lm(d[,i+214] ~ age.at.visit + sex.gp + digforct, data=d)

rr2 <- summary(fit2)

t2 <- (rr2$coefficients["digforct","t value"])

p2 <- (rr2$coefficients["digforct","Pr(>|t|)"])

e2 <- round(rr2$coefficients["digforct","Estimate"],digits=6)

se2 <- round(rr2$coefficients["digforct","Std. Error"],digits=6)

l2 <- confint(fit2, "digforct", level=0.95)[1]

u2 <- confint(fit2, "digforct", level=0.95)[2]

\## mdsupdrs3.total

fit4 <- lm(d[,i+214] ~ age.at.visit + sex.gp + mdsupdrs3.total, data=d)

rr4 <- summary(fit4)

t4 <- (rr4$coefficients["mdsupdrs3.total","t value"])

p4 <- (rr4$coefficients["mdsupdrs3.total","Pr(>|t|)"])

e4 <- round(rr4$coefficients["mdsupdrs3.total","Estimate"],digits=6)

se4 <- round(rr4$coefficients["mdsupdrs3.total","Std. Error"],digits=6)

l4 <- confint(fit4, "mdsupdrs3.total", level=0.95)[1]

u4 <- confint(fit4, "mdsupdrs3.total", level=0.95)[2]

\## scopa.aut.total

fit5 <- lm(d[,i+214] ~ age.at.visit + sex.gp + scopa.aut.total, data=d)

rr5 <- summary(fit5)

t5 <- (rr5$coefficients["scopa.aut.total","t value"])

p5 <- (rr5$coefficients["scopa.aut.total","Pr(>|t|)"])

e5 <- round(rr5$coefficients["scopa.aut.total","Estimate"],digits=6)

se5 <- round(rr5$coefficients["scopa.aut.total","Std. Error"],digits=6)

l5 <- confint(fit5, "scopa.aut.total", level=0.95)[1]

u5 <- confint(fit5, "scopa.aut.total", level=0.95)[2]

\## psrs.sum.score

fit6 <- lm(d[,i+214] ~ age.at.visit + sex.gp + psrs.sum.score, data=d)

rr6 <- summary(fit6)

t6 <- (rr6$coefficients["psrs.sum.score","t value"])

p6 <- (rr6$coefficients["psrs.sum.score","Pr(>|t|)"])

e6 <- round(rr6$coefficients["psrs.sum.score","Estimate"],digits=6)

se6 <- round(rr6$coefficients["psrs.sum.score","Std. Error"],digits=6)

l6 <- confint(fit6, "psrs.sum.score", level=0.95)[1]

u6 <- confint(fit6, "psrs.sum.score", level=0.95)[2]

\## epworth.total

fit7 <- lm(d[,i+214] ~ age.at.visit + sex.gp + epworth.total, data=d)

rr7 <- summary(fit7)

t7 <- (rr7$coefficients["epworth.total","t value"])

p7 <- (rr7$coefficients["epworth.total","Pr(>|t|)"])

e7 <- round(rr7$coefficients["epworth.total","Estimate"],digits=6)

se7 <- round(rr7$coefficients["epworth.total","Std. Error"],digits=6)

l7 <- confint(fit7, "epworth.total", level=0.95)[1]

u7 <- confint(fit7, "epworth.total", level=0.95)[2]

\## bsit

fit8 <- lm(d[,i+214] ~ age.at.visit + sex.gp + bsit, data=d)

rr8 <- summary(fit8)

t8 <- (rr8$coefficients["bsit","t value"])

p8 <- (rr8$coefficients["bsit","Pr(>|t|)"])

e8 <- round(rr8$coefficients["bsit","Estimate"],digits=6)

se8 <- round(rr8$coefficients["bsit","Std. Error"],digits=6)

l8 <- confint(fit8, "bsit", level=0.95)[1]

u8 <- confint(fit8, "bsit", level=0.95)[2]

\## pp.sum

fit9 <- lm(d[,i+214] ~ age.at.visit + sex.gp + pp.sum, data=d)

rr9 <- summary(fit9)

t9 <- (rr9$coefficients["pp.sum","t value"])

p9 <- (rr9$coefficients["pp.sum","Pr(>|t|)"])

e9 <- round(rr9$coefficients["pp.sum","Estimate"],digits=6)

se9 <- round(rr9$coefficients["pp.sum","Std. Error"],digits=6)

l9 <- confint(fit9, "pp.sum", level=0.95)[1]

u9 <- confint(fit9, "pp.sum", level=0.95)[2]

\##

reg1[i,1] <- colnames(aa[1])

reg1[i,2] <- t1

reg1[i,3] <- p1

reg1[i,4] <- l1

reg1[i,5] <- u1

reg1[i,6] <- e1

reg1[i,7] <- t2

reg1[i,8] <- p2

reg1[i,9] <- l2

reg1[i,10] <- u2

reg1[i,11] <- e2

reg1[i,12] <- t3

reg1[i,13] <- p3

reg1[i,14] <- l3

reg1[i,15] <- u3

reg1[i,16] <- e3

reg1[i,17] <- t4

reg1[i,18] <- p4

reg1[i,19] <- l4

reg1[i,20] <- u4

reg1[i,21] <- e4

reg1[i,22] <- t5

reg1[i,23] <- p5

reg1[i,24] <- l5

reg1[i,25] <- u5

reg1[i,26] <- e5

reg1[i,27] <- t6

reg1[i,28] <- p6

reg1[i,29] <- l6

reg1[i,30] <- u6

reg1[i,31] <- e6

reg1[i,32] <- t7

reg1[i,33] <- p7

reg1[i,34] <- l7

reg1[i,35] <- u7

reg1[i,36] <- e7

reg1[i,37] <- t8

reg1[i,38] <- p8

reg1[i,39] <- l8

reg1[i,40] <- u8

reg1[i,41] <- e8

reg1[i,42] <- t9

reg1[i,43] <- p9

reg1[i,44] <- l9

reg1[i,45] <- u9

reg1[i,46] <- e9

reg1[i,47] <- t10

reg1[i,48] <- p10

reg1[i,49] <- l10

reg1[i,50] <- u10

reg1[i,51] <- e10

rm(fit1,rr,t1,p1,e1,se1,l1,u1,fit2,rr2,t2,p2,e2,se2,l2,u2,fit3,rr3,t3,p3,e3,se3,l3,u3,fit4,rr4,t4,p4,e4,se4,l4,u4,fit5,rr5,t5,p5,e5,se5,l5,u5,fit6,rr6,t6,p6,e6,se6,l6,u6,fit7,rr7,t7,p7,e7,se7,l7,u7,fit8,rr8,t8,p8,e8,se8,l8,u8,fit9,rr9,t9,e9,se9,l9,u9,fit10,rr10,t10,p10,e10,se10,l10,u10)

}

head(reg1)

###############################################################

names(reg1)[1] <- "response"

names(reg1)[2] <- "moca.t"

names(reg1)[3] <- "moca.p"

names(reg1)[4] <- "moca.lcl"

names(reg1)[5] <- "moca.ucl"

names(reg1)[6] <- "moca.e"

names(reg1)[7] <- "dig.for"

names(reg1)[8] <- "dig.for.p"

names(reg1)[9] <- "dig.for.lcl"

names(reg1)[10] <- "dig.for.ucl"

names(reg1)[11] <- "dig.for.e"

names(reg1)[12] <- "dig.bac"

names(reg1)[13] <- "dig.bac.p"

names(reg1)[14] <- "dig.bac.lcl"

names(reg1)[15] <- "dig.bac.ucl"

names(reg1)[16] <- "dig.bac.e"

names(reg1)[17] <- "updrs"

names(reg1)[18] <- "updrs.p"

names(reg1)[19] <- "updrs.lcl"

names(reg1)[20] <- "updrs.ucl"

names(reg1)[21] <- "updrs.e"

names(reg1)[22] <- "scopa"

names(reg1)[23] <- "scopa.p"

names(reg1)[24] <- "scopa.lcl"

names(reg1)[25] <- "scopa.ucl"

names(reg1)[26] <- "scopa.e"

names(reg1)[27] <- "psrs"

names(reg1)[28] <- "psrs.p"

names(reg1)[29] <- "psrs.lcl"

names(reg1)[30] <- "psrs.ucl"

names(reg1)[31] <- "psrs.e"

names(reg1)[32] <- "epworth"

names(reg1)[33] <- "epworth.p"

names(reg1)[34] <- "epworth.lcl"

names(reg1)[35] <- "epworth.ucl"

names(reg1)[36] <- "epworth.e"

names(reg1)[37] <- "bsit"

names(reg1)[38] <- "bsit.p"

names(reg1)[39] <- "bsit.lcl"

names(reg1)[40] <- "bsit.ucl"

names(reg1)[41] <- "bsit.e"

names(reg1)[42] <- "purdue"

names(reg1)[43] <- "purdue.p"

names(reg1)[44] <- "purdue.lcl"

names(reg1)[45] <- "purdue.ucl"

names(reg1)[46] <- "purdue.e"

names(reg1)[47] <- "alt.tap"

names(reg1)[48] <- "alt.tap.p"

names(reg1)[49] <- "alt.tap.lcl"

names(reg1)[50] <- "alt.tap.ucl"

names(reg1)[51] <- "alt.tap.e"

\###

reg1$ROI <- toupper(gsub(".", " ", reg1$response, fixed=TRUE))

reg1$ROI2 <- toupper(gsub("fa", " ", reg1$ROI, fixed=TRUE))

reg1$ROI2 <- toupper(gsub("md", " ", reg1$ROI2, fixed=TRUE))

reg1$ROI2 <- toupper(gsub("tndi", " ", reg1$ROI2, fixed=TRUE))

reg1$ROI2 <- toupper(gsub("odi", " ", reg1$ROI2, fixed=TRUE))

reg1$ROI2 <- toupper(gsub("fwf", " ", reg1$ROI2, fixed=TRUE))

reg1$ROI2 <- toupper(gsub("ndi", " ", reg1$ROI2, fixed=TRUE))

\## MOCA regular p-value

reg1$d.moca <- ifelse(reg1$moca.p <0.05,"red","black")

reg1$p.moca <- pval(reg1$moca.p)

\## FDR adjustments

reg1$fdr.moca.p <- p.adjust(reg1$moca.p,method='fdr')

reg1$fdr.d.moca <- ifelse(reg1$fdr.moca.p <0.05,"red","black")

reg1$fdr.p.moca <- pval(reg1$fdr.moca.p)

\## digforct regular p-value

reg1$d.dig.for <- ifelse(reg1$dig.for.p <0.05,"red","black")

reg1$p.dig.for <- pval(reg1$dig.for.p)

\## FDR adjustments

reg1$fdr.dig.for.p <- p.adjust(reg1$dig.for.p,method='fdr')

reg1$fdr.d.dig.for <- ifelse(reg1$fdr.dig.for.p <0.05,"red","black")

reg1$fdr.p.dig.for <- pval(reg1$fdr.dig.for.p)

\## UPDRS

reg1$d.updrs <- ifelse(reg1$updrs.p <0.05,"red","black")

reg1$p.updrs <- pval(reg1$updrs.p)

\## FDR adjustments

reg1$fdr.updrs.p <- p.adjust(reg1$updrs.p,method='fdr')

reg1$fdr.d.updrs <- ifelse(reg1$fdr.updrs.p <0.05,"red","black")

reg1$fdr.p.updrs <- pval(reg1$fdr.updrs.p)

\## SCOPA

reg1$d.scopa <- ifelse(reg1$scopa.p <0.05,"red","black")

reg1$p.scopa <- pval(reg1$scopa.p)

\## FDR adjustments

reg1$fdr.scopa.p <- p.adjust(reg1$scopa.p,method='fdr')

reg1$fdr.d.scopa <- ifelse(reg1$fdr.scopa.p <0.05,"red","black")

reg1$fdr.p.scopa <- pval(reg1$fdr.scopa.p)

\## PSRS

reg1$d.psrs <- ifelse(reg1$psrs.p <0.05,"red","black")

reg1$p.psrs <- pval(reg1$psrs.p)

\## FDR adjustments

reg1$fdr.psrs.p <- p.adjust(reg1$psrs.p,method='fdr')

reg1$fdr.d.psrs <- ifelse(reg1$fdr.psrs.p <0.05,"red","black")

reg1$fdr.p.psrs <- pval(reg1$fdr.psrs.p)

\## EPWORTH

reg1$d.epworth <- ifelse(reg1$epworth.p <0.05,"red","black")

reg1$p.epworth <- pval(reg1$epworth.p)

\## FDR adjustments

reg1$fdr.epworth.p <- p.adjust(reg1$epworth.p,method='fdr')

reg1$fdr.d.epworth <- ifelse(reg1$fdr.epworth.p <0.05,"red","black")

reg1$fdr.p.epworth <- pval(reg1$fdr.epworth.p)

\## BSIT

reg1$d.bsit <- ifelse(reg1$bsit.p <0.05,"red","black")

reg1$p.bsit <- pval(reg1$bsit.p)

\## FDR adjustments

reg1$fdr.bsit.p <- p.adjust(reg1$bsit.p,method='fdr')

reg1$fdr.d.bsit <- ifelse(reg1$fdr.bsit.p <0.05,"red","black")

reg1$fdr.p.bsit <- pval(reg1$fdr.bsit.p)

\## purdue

reg1$d.purdue <- ifelse(reg1$purdue.p <0.05,"red","black")

reg1$p.purdue <- pval(reg1$purdue.p)

\## FDR adjustments

reg1$fdr.purdue.p <- p.adjust(reg1$purdue.p,method='fdr')

reg1$fdr.d.purdue <- ifelse(reg1$fdr.purdue.p <0.05,"red","black")

reg1$fdr.p.purdue <- pval(reg1$fdr.purdue.p)

\## Alt.TAP

reg1$d.alt.tap <- ifelse(reg1$alt.tap.p <0.05,"red","black")

reg1$p.alt.tap <- pval(reg1$alt.tap.p)

\## FDR adjustments

reg1$fdr.alt.tap.p <- p.adjust(reg1$alt.tap.p,method='fdr')

reg1$fdr.d.alt.tap <- ifelse(reg1$fdr.alt.tap.p <0.05,"red","black")

reg1$fdr.p.alt.tap <- pval(reg1$fdr.alt.tap.p)

cfa <- reg1[1:39,]

cmd <- reg1[40:78,]

ctndi <- reg1[79:117,]

codi <- reg1[118:156,]

cfwf <- reg1[157:195,]

cndi <- reg1[196:234,]

\## redo the fdr for each

\## MOCA FDR adjustments

cfa$fdr.moca.p <- p.adjust(cfa$moca.p,method='fdr')

cfa$fdr.d.moca <- ifelse(cfa$fdr.moca.p <0.05,"red","black")

cfa$fdr.p.moca <- pval(cfa$fdr.moca.p)

\## dig.for

\## FDR adjustments

cfa$fdr.dig.for.p <- p.adjust(cfa$dig.for.p,method='fdr')

cfa$fdr.d.dig.for <- ifelse(cfa$fdr.dig.for.p <0.05,"red","black")

cfa$fdr.p.dig.for <- pval(cfa$fdr.dig.for.p)

\## dig.back

\## FDR adjustments

cfa$fdr.dig.bac.p <- p.adjust(cfa$dig.bac.p,method='fdr')

cfa$fdr.d.dig.bac <- ifelse(cfa$fdr.dig.bac.p <0.05,"red","black")

cfa$fdr.p.dig.bac <- pval(cfa$fdr.dig.bac.p)

\## UPDRS

\## FDR adjustments

cfa$fdr.updrs.p <- p.adjust(cfa$updrs.p,method='fdr')

cfa$fdr.d.updrs <- ifelse(cfa$fdr.updrs.p <0.05,"red","black")

cfa$fdr.p.updrs <- pval(cfa$fdr.updrs.p)

\## SCOPA

\## FDR adjustments

cfa$fdr.scopa.p <- p.adjust(cfa$scopa.p,method='fdr')

cfa$fdr.d.scopa <- ifelse(cfa$fdr.scopa.p <0.05,"red","black")

cfa$fdr.p.scopa <- pval(cfa$fdr.scopa.p)

\## PSRS

\## FDR adjustments

cfa$fdr.psrs.p <- p.adjust(cfa$psrs.p,method='fdr')

cfa$fdr.d.psrs <- ifelse(cfa$fdr.psrs.p <0.05,"red","black")

cfa$fdr.p.psrs <- pval(cfa$fdr.psrs.p)

\## EPWORTH

\## FDR adjustments

cfa$fdr.epworth.p <- p.adjust(cfa$epworth.p,method='fdr')

cfa$fdr.d.epworth <- ifelse(cfa$fdr.epworth.p <0.05,"red","black")

cfa$fdr.p.epworth <- pval(cfa$fdr.epworth.p)

\## BSIT

\## FDR adjustments

cfa$fdr.bsit.p <- p.adjust(cfa$bsit.p,method='fdr')

cfa$fdr.d.bsit <- ifelse(cfa$fdr.bsit.p <0.05,"red","black")

cfa$fdr.p.bsit <- pval(cfa$fdr.bsit.p)

\## PURDUE

\## FDR adjustments

cfa$fdr.purdue.p <- p.adjust(cfa$purdue.p,method='fdr')

cfa$fdr.d.purdue <- ifelse(cfa$fdr.purdue.p <0.05,"red","black")

cfa$fdr.p.purdue <- pval(cfa$fdr.purdue.p)

\## ALT.TAP

\## FDR adjustments

cfa$fdr.alt.tap.p <- p.adjust(cfa$alt.tap.p,method='fdr')

cfa$fdr.d.alt.tap <- ifelse(cfa$fdr.alt.tap.p <0.05,"red","black")

cfa$fdr.p.alt.tap <- pval(cfa$fdr.alt.tap.p)

\## CMD

\## MOCA FDR adjustments

cmd$fdr.moca.p <- p.adjust(cmd$moca.p,method='fdr')

cmd$fdr.d.moca <- ifelse(cmd$fdr.moca.p <0.05,"red","black")

cmd$fdr.p.moca <- pval(cmd$fdr.moca.p)

\## dig.for

\## FDR adjustments

cmd$fdr.dig.for.p <- p.adjust(cmd$dig.for.p,method='fdr')

cmd$fdr.d.dig.for <- ifelse(cmd$fdr.dig.for.p <0.05,"red","black")

cmd$fdr.p.dig.for <- pval(cmd$fdr.dig.for.p)

\## dig.back

\## FDR adjustments

cmd$fdr.dig.bac.p <- p.adjust(cmd$dig.bac.p,method='fdr')

cmd$fdr.d.dig.bac <- ifelse(cmd$fdr.dig.bac.p <0.05,"red","black")

cmd$fdr.p.dig.bac <- pval(cmd$fdr.dig.bac.p)

\## UPDRS

\## FDR adjustments

cmd$fdr.updrs.p <- p.adjust(cmd$updrs.p,method='fdr')

cmd$fdr.d.updrs <- ifelse(cmd$fdr.updrs.p <0.05,"red","black")

cmd$fdr.p.updrs <- pval(cmd$fdr.updrs.p)

\## SCOPA

\## FDR adjustments

cmd$fdr.scopa.p <- p.adjust(cmd$scopa.p,method='fdr')

cmd$fdr.d.scopa <- ifelse(cmd$fdr.scopa.p <0.05,"red","black")

cmd$fdr.p.scopa <- pval(cmd$fdr.scopa.p)

\## PSRS

\## FDR adjustments

cmd$fdr.psrs.p <- p.adjust(cmd$psrs.p,method='fdr')

cmd$fdr.d.psrs <- ifelse(cmd$fdr.psrs.p <0.05,"red","black")

cmd$fdr.p.psrs <- pval(cmd$fdr.psrs.p)

\## EPWORTH

\## FDR adjustments

cmd$fdr.epworth.p <- p.adjust(cmd$epworth.p,method='fdr')

cmd$fdr.d.epworth <- ifelse(cmd$fdr.epworth.p <0.05,"red","black")

cmd$fdr.p.epworth <- pval(cmd$fdr.epworth.p)

\## BSIT

\## FDR adjustments

cmd$fdr.bsit.p <- p.adjust(cmd$bsit.p,method='fdr')

cmd$fdr.d.bsit <- ifelse(cmd$fdr.bsit.p <0.05,"red","black")

cmd$fdr.p.bsit <- pval(cmd$fdr.bsit.p)

\## PURDUE

\## FDR adjustments

cmd$fdr.purdue.p <- p.adjust(cmd$purdue.p,method='fdr')

cmd$fdr.d.purdue <- ifelse(cmd$fdr.purdue.p <0.05,"red","black")

cmd$fdr.p.purdue <- pval(cmd$fdr.purdue.p)

\## ALT.TAP

\## FDR adjustments

cmd$fdr.alt.tap.p <- p.adjust(cmd$alt.tap.p,method='fdr')

cmd$fdr.d.alt.tap <- ifelse(cmd$fdr.alt.tap.p <0.05,"red","black")

cmd$fdr.p.alt.tap <- pval(cmd$fdr.alt.tap.p)

\## ctndi

\## MOCA FDR adjustments

ctndi$fdr.moca.p <- p.adjust(ctndi$moca.p,method='fdr')

ctndi$fdr.d.moca <- ifelse(ctndi$fdr.moca.p <0.05,"red","black")

ctndi$fdr.p.moca <- pval(ctndi$fdr.moca.p)

\## dig.for

\## FDR adjustments

ctndi$fdr.dig.for.p <- p.adjust(ctndi$dig.for.p,method='fdr')

ctndi$fdr.d.dig.for <- ifelse(ctndi$fdr.dig.for.p <0.05,"red","black")

ctndi$fdr.p.dig.for <- pval(ctndi$fdr.dig.for.p)

\## dig.back

\## FDR adjustments

ctndi$fdr.dig.bac.p <- p.adjust(ctndi$dig.bac.p,method='fdr')

ctndi$fdr.d.dig.bac <- ifelse(ctndi$fdr.dig.bac.p <0.05,"red","black")

ctndi$fdr.p.dig.bac <- pval(ctndi$fdr.dig.bac.p)

\## UPDRS

\## FDR adjustments

ctndi$fdr.updrs.p <- p.adjust(ctndi$updrs.p,method='fdr')

ctndi$fdr.d.updrs <- ifelse(ctndi$fdr.updrs.p <0.05,"red","black")

ctndi$fdr.p.updrs <- pval(ctndi$fdr.updrs.p)

\## SCOPA

\## FDR adjustments

ctndi$fdr.scopa.p <- p.adjust(ctndi$scopa.p,method='fdr')

ctndi$fdr.d.scopa <- ifelse(ctndi$fdr.scopa.p <0.05,"red","black")

ctndi$fdr.p.scopa <- pval(ctndi$fdr.scopa.p)

\## PSRS

\## FDR adjustments

ctndi$fdr.psrs.p <- p.adjust(ctndi$psrs.p,method='fdr')

ctndi$fdr.d.psrs <- ifelse(ctndi$fdr.psrs.p <0.05,"red","black")

ctndi$fdr.p.psrs <- pval(ctndi$fdr.psrs.p)

\## EPWORTH

\## FDR adjustments

ctndi$fdr.epworth.p <- p.adjust(ctndi$epworth.p,method='fdr')

ctndi$fdr.d.epworth <- ifelse(ctndi$fdr.epworth.p <0.05,"red","black")

ctndi$fdr.p.epworth <- pval(ctndi$fdr.epworth.p)

\## BSIT

\## FDR adjustments

ctndi$fdr.bsit.p <- p.adjust(ctndi$bsit.p,method='fdr')

ctndi$fdr.d.bsit <- ifelse(ctndi$fdr.bsit.p <0.05,"red","black")

ctndi$fdr.p.bsit <- pval(ctndi$fdr.bsit.p)

\## PURDUE

\## FDR adjustments

ctndi$fdr.purdue.p <- p.adjust(ctndi$purdue.p,method='fdr')

ctndi$fdr.d.purdue <- ifelse(ctndi$fdr.purdue.p <0.05,"red","black")

ctndi$fdr.p.purdue <- pval(ctndi$fdr.purdue.p)

\## ALT.TAP

\## FDR adjustments

ctndi$fdr.alt.tap.p <- p.adjust(ctndi$alt.tap.p,method='fdr')

ctndi$fdr.d.alt.tap <- ifelse(ctndi$fdr.alt.tap.p <0.05,"red","black")

ctndi$fdr.p.alt.tap <- pval(ctndi$fdr.alt.tap.p)

\## codi

\## MOCA FDR adjustments

codi$fdr.moca.p <- p.adjust(codi$moca.p,method='fdr')

codi$fdr.d.moca <- ifelse(codi$fdr.moca.p <0.05,"red","black")

codi$fdr.p.moca <- pval(codi$fdr.moca.p)

\## dig.for

\## FDR adjustments

codi$fdr.dig.for.p <- p.adjust(codi$dig.for.p,method='fdr')

codi$fdr.d.dig.for <- ifelse(codi$fdr.dig.for.p <0.05,"red","black")

codi$fdr.p.dig.for <- pval(codi$fdr.dig.for.p)

\## dig.back

\## FDR adjustments

codi$fdr.dig.bac.p <- p.adjust(codi$dig.bac.p,method='fdr')

codi$fdr.d.dig.bac <- ifelse(codi$fdr.dig.bac.p <0.05,"red","black")

codi$fdr.p.dig.bac <- pval(codi$fdr.dig.bac.p)

\## UPDRS

\## FDR adjustments

codi$fdr.updrs.p <- p.adjust(codi$updrs.p,method='fdr')

codi$fdr.d.updrs <- ifelse(codi$fdr.updrs.p <0.05,"red","black")

codi$fdr.p.updrs <- pval(codi$fdr.updrs.p)

\## SCOPA

\## FDR adjustments

codi$fdr.scopa.p <- p.adjust(codi$scopa.p,method='fdr')

codi$fdr.d.scopa <- ifelse(codi$fdr.scopa.p <0.05,"red","black")

codi$fdr.p.scopa <- pval(codi$fdr.scopa.p)

\## PSRS

\## FDR adjustments

codi$fdr.psrs.p <- p.adjust(codi$psrs.p,method='fdr')

codi$fdr.d.psrs <- ifelse(codi$fdr.psrs.p <0.05,"red","black")

codi$fdr.p.psrs <- pval(codi$fdr.psrs.p)

\## EPWORTH

\## FDR adjustments

codi$fdr.epworth.p <- p.adjust(codi$epworth.p,method='fdr')

codi$fdr.d.epworth <- ifelse(codi$fdr.epworth.p <0.05,"red","black")

codi$fdr.p.epworth <- pval(codi$fdr.epworth.p)

\## BSIT

\## FDR adjustments

codi$fdr.bsit.p <- p.adjust(codi$bsit.p,method='fdr')

codi$fdr.d.bsit <- ifelse(codi$fdr.bsit.p <0.05,"red","black")

codi$fdr.p.bsit <- pval(codi$fdr.bsit.p)

\## PURDUE

\## FDR adjustments

codi$fdr.purdue.p <- p.adjust(codi$purdue.p,method='fdr')

codi$fdr.d.purdue <- ifelse(codi$fdr.purdue.p <0.05,"red","black")

codi$fdr.p.purdue <- pval(codi$fdr.purdue.p)

\## ALT.TAP

\## FDR adjustments

codi$fdr.alt.tap.p <- p.adjust(codi$alt.tap.p,method='fdr')

codi$fdr.d.alt.tap <- ifelse(codi$fdr.alt.tap.p <0.05,"red","black")

codi$fdr.p.alt.tap <- pval(codi$fdr.alt.tap.p)

\## cfwf

\## MOCA FDR adjustments

cfwf$fdr.moca.p <- p.adjust(cfwf$moca.p,method='fdr')

cfwf$fdr.d.moca <- ifelse(cfwf$fdr.moca.p <0.05,"red","black")

cfwf$fdr.p.moca <- pval(cfwf$fdr.moca.p)

\## dig.for

\## FDR adjustments

cfwf$fdr.dig.for.p <- p.adjust(cfwf$dig.for.p,method='fdr')

cfwf$fdr.d.dig.for <- ifelse(cfwf$fdr.dig.for.p <0.05,"red","black")

cfwf$fdr.p.dig.for <- pval(cfwf$fdr.dig.for.p)

\## dig.back

\## FDR adjustments

cfwf$fdr.dig.bac.p <- p.adjust(cfwf$dig.bac.p,method='fdr')

cfwf$fdr.d.dig.bac <- ifelse(cfwf$fdr.dig.bac.p <0.05,"red","black")

cfwf$fdr.p.dig.bac <- pval(cfwf$fdr.dig.bac.p)

\## UPDRS

\## FDR adjustments

cfwf$fdr.updrs.p <- p.adjust(cfwf$updrs.p,method='fdr')

cfwf$fdr.d.updrs <- ifelse(cfwf$fdr.updrs.p <0.05,"red","black")

cfwf$fdr.p.updrs <- pval(cfwf$fdr.updrs.p)

\## SCOPA

\## FDR adjustments

cfwf$fdr.scopa.p <- p.adjust(cfwf$scopa.p,method='fdr')

cfwf$fdr.d.scopa <- ifelse(cfwf$fdr.scopa.p <0.05,"red","black")

cfwf$fdr.p.scopa <- pval(cfwf$fdr.scopa.p)

\## PSRS

\## FDR adjustments

cfwf$fdr.psrs.p <- p.adjust(cfwf$psrs.p,method='fdr')

cfwf$fdr.d.psrs <- ifelse(cfwf$fdr.psrs.p <0.05,"red","black")

cfwf$fdr.p.psrs <- pval(cfwf$fdr.psrs.p)

\## EPWORTH

\## FDR adjustments

cfwf$fdr.epworth.p <- p.adjust(cfwf$epworth.p,method='fdr')

cfwf$fdr.d.epworth <- ifelse(cfwf$fdr.epworth.p <0.05,"red","black")

cfwf$fdr.p.epworth <- pval(cfwf$fdr.epworth.p)

\## BSIT

\## FDR adjustments

cfwf$fdr.bsit.p <- p.adjust(cfwf$bsit.p,method='fdr')

cfwf$fdr.d.bsit <- ifelse(cfwf$fdr.bsit.p <0.05,"red","black")

cfwf$fdr.p.bsit <- pval(cfwf$fdr.bsit.p)

\## PURDUE

\## FDR adjustments

cfwf$fdr.purdue.p <- p.adjust(cfwf$purdue.p,method='fdr')

cfwf$fdr.d.purdue <- ifelse(cfwf$fdr.purdue.p <0.05,"red","black")

cfwf$fdr.p.purdue <- pval(cfwf$fdr.purdue.p)

\## ALT.TAP

\## FDR adjustments

cfwf$fdr.alt.tap.p <- p.adjust(cfwf$alt.tap.p,method='fdr')

cfwf$fdr.d.alt.tap <- ifelse(cfwf$fdr.alt.tap.p <0.05,"red","black")

cfwf$fdr.p.alt.tap <- pval(cfwf$fdr.alt.tap.p)

\## cndi

\## MOCA FDR adjustments

cndi$fdr.moca.p <- p.adjust(cndi$moca.p,method='fdr')

cndi$fdr.d.moca <- ifelse(cndi$fdr.moca.p <0.05,"red","black")

cndi$fdr.p.moca <- pval(cndi$fdr.moca.p)

\## dig.for

\## FDR adjustments

cndi$fdr.dig.for.p <- p.adjust(cndi$dig.for.p,method='fdr')

cndi$fdr.d.dig.for <- ifelse(cndi$fdr.dig.for.p <0.05,"red","black")

cndi$fdr.p.dig.for <- pval(cndi$fdr.dig.for.p)

\## dig.back

\## FDR adjustments

cndi$fdr.dig.bac.p <- p.adjust(cndi$dig.bac.p,method='fdr')

cndi$fdr.d.dig.bac <- ifelse(cndi$fdr.dig.bac.p <0.05,"red","black")

cndi$fdr.p.dig.bac <- pval(cndi$fdr.dig.bac.p)

\## UPDRS

\## FDR adjustments

cndi$fdr.updrs.p <- p.adjust(cndi$updrs.p,method='fdr')

cndi$fdr.d.updrs <- ifelse(cndi$fdr.updrs.p <0.05,"red","black")

cndi$fdr.p.updrs <- pval(cndi$fdr.updrs.p)

\## SCOPA

\## FDR adjustments

cndi$fdr.scopa.p <- p.adjust(cndi$scopa.p,method='fdr')

cndi$fdr.d.scopa <- ifelse(cndi$fdr.scopa.p <0.05,"red","black")

cndi$fdr.p.scopa <- pval(cndi$fdr.scopa.p)

\## PSRS

\## FDR adjustments

cndi$fdr.psrs.p <- p.adjust(cndi$psrs.p,method='fdr')

cndi$fdr.d.psrs <- ifelse(cndi$fdr.psrs.p <0.05,"red","black")

cndi$fdr.p.psrs <- pval(cndi$fdr.psrs.p)

\## EPWORTH

\## FDR adjustments

cndi$fdr.epworth.p <- p.adjust(cndi$epworth.p,method='fdr')

cndi$fdr.d.epworth <- ifelse(cndi$fdr.epworth.p <0.05,"red","black")

cndi$fdr.p.epworth <- pval(cndi$fdr.epworth.p)

\## BSIT

\## FDR adjustments

cndi$fdr.bsit.p <- p.adjust(cndi$bsit.p,method='fdr')

cndi$fdr.d.bsit <- ifelse(cndi$fdr.bsit.p <0.05,"red","black")

cndi$fdr.p.bsit <- pval(cndi$fdr.bsit.p)

\## PURDUE

\## FDR adjustments

cndi$fdr.purdue.p <- p.adjust(cndi$purdue.p,method='fdr')

cndi$fdr.d.purdue <- ifelse(cndi$fdr.purdue.p <0.05,"red","black")

cndi$fdr.p.purdue <- pval(cndi$fdr.purdue.p)

\## ALT.TAP

\## FDR adjustments

cndi$fdr.alt.tap.p <- p.adjust(cndi$alt.tap.p,method='fdr')

cndi$fdr.d.alt.tap <- ifelse(cndi$fdr.alt.tap.p <0.05,"red","black")

cndi$fdr.p.alt.tap <- pval(cndi$fdr.alt.tap.p)
